# Supplementary material for: Rotavirus vaccine impact assessment surveillance in India: protocol and methods
Source: BMJ Open. 2019 Apr 25;9(4):e024840. doi: 10.1136/bmjopen-2018-024840 (PMC6502045; doi:10.1136/bmjopen-2018-024840)
Supplement: Supplementary file 1 [file bmjopen-2018-024840supp001.pdf]

SupplementaryTable1. Baseline data from sentinel surveillance sites included in the impact assessment project

Sl. no.	Name of surveillance site	No. of under- five AGE admissions a year#
1	Kurnool Medical College, Kurnool, Andhra Pradesh	410
2	Government General hospital, Kakinada, Andhra Pradesh	400
3	King George Hospital, Vishakhapatnam, Andhra Pradesh	300
4	Sri Venkateswara Medical College, Tirupati, AP	400
5	Sardar Vallabhai Patel Post Graduate Institute of Pediatrics, Cuttack, Odisha	720
6	Kalinga Institute of Medical Sciences, Bhubaneshwar, Odisha	340
7	Institute of Medical Sciences and SUM Hospital, Bhubaneshwar, Odisha	335
8	Hi-Tech Hospital, Bhubaneshwar, Odisha	490
9	Pandit Bhagwat Dayal Sharma Post Graduate Institute of Medical Sciences, Rohtak, Haryana	250
10	Shaheed Hasan Khan Mewati Government Medical College, Mewat, Haryana	620
11	BPS Government Medical College for Women, Sonipat, Haryana	320
12	Indira Gandhi Government Medical College, Shimla, HP	200
13	Rajendra Prasad Government Medical College, Tanda, HP	300
14	Post Graduate Institute of Medical Education and Research, Chandigarh	600

15	The Institute of Child Health and Hospital for Children, Chennai, Tamil Nadu	488
16	Kanchi Kama Koti Child Trust Hospital, Chennai, Tamil Nadu	528
17	Government Medical College, Madurai, Tamil Nadu	110
18	Christian Medical College, Vellore, Tamil Nadu	
19	Nalam Hospital, Vellore, Tamil Nadu	
20	Narayani Hospital and Research Centre, Vellore, Tamil Nadu	
21	GVMC, Adukkumparai, Tamil Nadu	
22	Sawai Man Singh Medical College, Jaipur, Rajasthan	1763
23	Dr. Sampurnanand Medical College, Jodhpur, Rajasthan	1627
24	Rabindranath Tagore Medical college, Udaipur, Rajasthan	1660
25	King George Medical College, Lucknow, Uttar Pradesh	244
26	Institute of Medical Sciences, Banaras Hindu University, Varanasi, Uttar Pradesh	144
27	BRD Medical College, Gorakhpur, Uttar Pradesh	600
28	Mangala Hospital and Research Centre, Bijnor, Uttar Pradesh	550
29	NSCB Medical College, Jabalpur, Madhya Pradesh	700
30	Mahatma Gandhi Memorial Medical college, Indore, Madhya Pradesh	710
31	Government Medical college, Guwahati, Assam	560
32	Baptist Christian Hospital, Tezpur, Assam	232

#Retrospective data for the year 2015
