# Supplementary material for: Rotavirus vaccine impact assessment surveillance in India: protocol and methods
Source: BMJ Open. 2019 Apr 25;9(4):e024840. doi: 10.1136/bmjopen-2018-024840 (PMC6502045; doi:10.1136/bmjopen-2018-024840)
Supplement: Supplementary file 2 [file bmjopen-2018-024840supp002.pdf]

Supplementary Table 2: Ethical committee details for Protocol submission

Sl. No.	Name of the institution	Name of the ethical committee (Reference number)
1	Kurnool Medical College, Kurnool, Andhra Pradesh	Institutional Ethical committee of Kurnool Medical College, Kurnool dated 12.11.2015
2	Government General Hospital, Kakinada, Andhra Pradesh	Institutional Ethics Committee cell of Rangaraya Medical College (IEC/RMC/2017/241 dated 06.05.2017)
3	Andhra Medical College, Vishakhapatnam, Andhra Pradesh	Institutional Ethical committee of King George Hospital, Vishakhapatnam (proposal 1 dated 19.11.2016)
4	Sri Venkateswara Medical College, Tirupati, Andhra Pradesh	Institutional Ethical committee of S.V Medical College (Lr. No. 2/2016 dated 07.02.2016)
5	Sardar Vallabhai Patel Post Graduate Institute of Pediatrics, Cuttack, Odisha	Institutional Ethical committee of SCB Medical College, Cuttack (IEC no. 273 dated 4.4.2016)
6	Kalinga Institute of Medical Sciences, Bhubaneswar, Odisha	Ethics committee of Kalinga Institute of Medical Sciences, Bhubaneswar (KIMS/Dir/11/16 dated 6.01.2016)
7	Institute of Medical Sciences and SUM Hospital, Bhubaneswar, Odisha	Institutional Ethical Committee of Institute of Medical Sciences and SUM Hospital, Bhubaneswar (Letter no. 232/20/11/2015 dated 20.11.2015)
8	Hi-Tech Hospital and Medical college, Bhubaneswar, Odisha	Institutional Ethical Committee of Hi-Tech Medical College and Hospital (HMCH/IEC/15/3067 dated 29.12.2015)
9	Pandit Bhagwat Dayal Sharma Post Graduate Institute of Medical Sciences, Rohtak, Haryana	Institutional Ethics Committee, Pandit Bhagwat Dayal Sharma Post Graduate Institute of

		Medical Sciences, Rohtak (No.IEC/16/41 dated 28.03.2016)
10	Shaheed Hasan Khan Mewati Government Medical College, Mewat, Haryana	Institutional Ethics Committee, Shaheed Hasan Khan Mewati Government Medical College, Mewat (SHKM/CM/2016/890 dated 13.02.2016)
11	BPS Government Medical College for Women, Sonipat, Haryana	Institutional Ethics Committee, BPS Government Medical College for Women, Sonipat (No. Peds/BPSGMC/16 dated 01.04.2016)
12	Post Graduate Institute of Medical Education and Research, Chandigarh	Institutional Ethics Committee, Post Graduate Institute of Medical Education and Research, Chandigarh (PGI/IEC/2016/3267 dated 03.08.2016)
13	Rajendra Prasad Government Medical College, Tanda, Himachal Pradesh	Institutional Ethics Committee, Rajendra Prasad Government Medical College, Tanda (HFW-H-DRPGMC/Ethics/2016 dated 21.01.2016)
14	Indira Gandhi Government Medical College, Shimla, Himachal Pradesh	Institutional Ethics Committee, Indira Gandhi Government Medical College, Shimla (ECR/533/Inst/HP/2014 dated 13.05.2016)
15	Kanchi Kama Koti Child Trust hospital, Chennai, Tamil Nadu	Ethics committee of Kanchi Kama Koti Childs Trust Hospital and the Childs Trust (CTMRF/EC/S/02/2017 dated 18.03.2017)
16	Institute of Child health, Egmore, Tamil Nadu	Institutional Ethics Committee of Madras Medical College, Chennai (No. 01052017 dated 02.05.2017)

17	Government Medical College, Madurai, Tamil Nadu	Ethics Committee of Madurai Medical College, Madurai (dated 27.11.2017)
18	Christian Medical College, Vellore, Tamil Nadu	Institutional Review Board, Christian Medical College, Vellore (IRB Min. no. 9530 dated 22.07.2015)
19	Government Vellore Medical College, Vellore, Tamil Nadu	Institutional Review Board, Christian Medical College, Vellore (IRB Min. no. 9530 dated 22.07.2015)
20	Nalam Hospital, Vellore, Tamil Nadu	Institutional Review Board, Christian Medical College, Vellore (IRB Min. no. 9530 dated 22.07.2015)
21	Narayani Hospital and Research Centre, Vellore, Tamil Nadu	Institutional Review Board, Christian Medical College, Vellore (IRB Min. no. 9530 dated 22.07.2015)
22	Sawai Man Singh Medical college, Jaipur, Rajasthan	Office of the Ethics Committee, Sawai Man Singh Medical college, Jaipur (3205/MC/EC/2017 dated 08.06.2017)
23	Rabindranath Tagore Medical College, Udaipur, Rajasthan	Ethics Committee, Rabindranath Tagore Medical College, Udaipur (RNT/STAT/IEC/2017/54 dated 18.04.2017)
24	Dr. Sampurnanand Medical College, Jodhpur, Rajasthan	Institutional Ethics Committee, Dr. Sampurnanand Medical College, Jodhpur (No.F.1/Acad/MC/JU/17/16277 dated 20.06.2017)
25	Mahatma Gandhi Memorial Medical College, Indore, Madhya Pradesh	Institutional Ethics Committee, Mahatma Gandhi Memorial

		Medical College, Indore (dated 24.05.2017)
26	Netaji Subhash Chandra Bose Medical College, Jabalpur, Madhya Pradesh	Institutional Review Board, Netaji Subhash Chandra Bose Medical College, Jabalpur, Madhya Pradesh
27	Baptist Christian Hospital, Tezpur, Assam	Institutional Ethics Committee, Emmanuel Hospital Association, New Delhi (Protocol No. 163 dated 23.03.2018)
28	Government Medical college, Guwahati, Assam	Institutional Review Board, Government Medical college, Guwahati (dated 28.02.2018)
29	King George Medical College, Lucknow, Uttar Pradesh	Institutional Ethics Committee, King George Medical University, Lucknow (No.515/Ethics/R-cell-17 dated 26.05.2017)
30	Institute of Medical Sciences, Banaras Hindu University, Varanasi, Uttar Pradesh	Institutional Ethics Committee, Institute of Medical Sciences, Banaras Hindu University, Varanasi (Dean/2016-17/EC/734 dated 31.03.2017)
31	Baba Ram Das Medical College, Gorakhpur, Uttar Pradesh	Institutional Review Board, Baba Ram Das Medical College, Gorakhpur, Uttar Pradesh (dated 20.07.2018)
32	Mangala Hospital and Research Centre, Bijnor, Uttar Pradesh	Institutional Ethics Committee, Mangala Hospital and Research Centre, Bijnor (IEC/MH/2018/01 dated 24.01.2018)
