# Supplementary material for: Rotavirus vaccine impact assessment surveillance in India: protocol and methods
Source: BMJ Open. 2019 Apr 25;9(4):e024840. doi: 10.1136/bmjopen-2018-024840 (PMC6502045; doi:10.1136/bmjopen-2018-024840)
Supplement: Supplementary file 3 [file bmjopen-2018-024840supp003.pdf]

Supplementary table 3: Monitoring checklist for sites doing under five diarrhea surveillance

SITE PROFILE

1. Staff

Name of staff
Position Title or Duties
Full-time or Part-time for rotavirus responsibilities
Percentage of time spent working on rotavirus
Years of experience in Site
Undergone training at CMC or at site

2. Staff and equipment

Designated place for project staff
Storage of records (ICF/CRF etc.)
Refrigerator for storage of stool samples

3. Project management and supervision

Lines of supervision and accountability clear to all staff
Written protocols are available, and arrangements are in place for periodic review and evaluation
Arrangements are made for back-up staff to perform work during staff absences

PROJECT ACTIVITIES

1. Recording of recruitment

Number of children admitted for gastroenteritis
Source from which the details in the hospital logbook like ward registers, online information, emergency records etc.
How many locations are children recruited from?

Number of children enrolled for the project so far (Informed consent form completed)
Reasons for non-enrolment
Number of children for whom case report form was filled
Reasons for non-completion of CRF
Number of children for whom stool sample was collected
Reasons for non-collection of stool sample
Problems encountered by the site in completing this information

2. Review of informed consent forms

Number of ICFs reviewed
Location of filing the forms
Legibility of the consent forms especially name, date and signature
Signatures of parents/guardians
Signature of the investigator
Problems encountered by the site in completing this information

3. Completion of Case Report Forms

Number of CRFs reviewed
Number of CRFs with missing data fields
Signature and a date for completion of CRF
Record of stool sample collection
Verification of stool sample collection against the hospital logbook
Verification of stool sample collection against the sample transmittal log

Problems encountered by the site in completing this information

4. Collection of vaccination information

What are the sources of vaccination information in the CRFs
Verification of dates on the CRFs from the source
Problems encountered by the site in completing this information

5. Collection of stool samples

Collection of stool samples
Number of samples collected
Labelling of the containers
Storage of samples in the deep freezer including temperature maintenance and positioning of the samples
Quantity of the collected samples
Verification of stool samples against the hospital logbook
Verification of stool samples against the CRF
Verification of stool sample collection against the sample transmittal log
Problems encountered by the site in completing this information

SUMMARY INDICATORS

Enrolment of >90% of admitted cases of gastroenteritis
Collection of adequate stools from >90% of enrolled children
Complete documentation (ICF/CRF) in >90% of enrolled children
Collection of vaccination record (patient retained/government) for >75% of enrolled children
