# Supplementary material for: Rotavirus vaccine impact assessment surveillance in India: protocol and methods
Source: BMJ Open. 2019 Apr 25;9(4):e024840. doi: 10.1136/bmjopen-2018-024840 (PMC6502045; doi:10.1136/bmjopen-2018-024840)
Supplement: Author's manuscript [file bmjopen-2018-024840.draft_revisions.pdf]

BMJ Open

BMJ Open is committed to open peer review. As part of this commitment we make the peer review history of every article we publish publicly available.

When an article is published we post the peer reviewers' comments and the authors' responses online. We also post the versions of the paper that were used during peer review. These are the versions that the peer review comments apply to.

The versions of the paper that follow are the versions that were submitted during the peer review process. They are not the versions of record or the final published versions. They should not be cited or distributed as the published version of this manuscript.

BMJ Open is an open access journal and the full, final, typeset and author-corrected version of record of the manuscript is available on our site with no access controls, subscription charges or pay-per-view fees (http://bmjopen.bmj.com).

If you have any questions on BMJ Open's open peer review process please email info.bmjopen@bmj.com

BMJ Open

Rotavirus vaccine impact assessment surveillance in India: Protocol and methods

Journal:	*BMJ Open*
Manuscript ID	bmjopen-2018-024840
Article Type:	Protocol
Date Submitted by the Author:	16-Jun-2018
Complete List of Authors:	Nair, Nayana; Christian Medical College, Vellore, Department of GI Sciences<br>Reddy, Samarasimha; Christian Medical College, Vellore, Department of GI Sciences<br>Giri, Sidhartha; Christian Medical College, Vellore, Department of GI Sciences<br>Mohan, Venkata Raghava; Christian Medical College ,<br>Parashar, Umesh; Centers for Disease Control and Prevention, Atlanta<br>Tate, Jacqueline; Centers for Disease Control and Prevention<br>Shah, Minesh; Centers for Disease Control and Prevention<br>Arora, Rashmi; Indian Council of Medical Research<br>Gupte, Mohan; Indian Council of Medical Research<br>Mehendale, Sanjay; National Institute of Epidemiology<br>Kang, Gagandeep; Christian Medical College, Vellore, Department of GI Sciences; Translational Health Science and Technology Institute, Faridabad
Keywords:	Rotavirus, Surveillance, India, vaccine, impact

SCHOLARONE™
Manuscripts

Rotavirus vaccine impact assessment surveillance in India: Protocol and methods

Authors

Nayana P Nair[1], Samarasimha Reddy N[1], Sidhartha Giri[1], Venkata Raghava Mohan[2], Umesh D. Parashar[3], Jacqueline E. Tate[3], Minesh Pradyuman Shah[3], Rashmi Arora[4], M D Gupte[4], Sanjay Mehendale[5], Investigators of the rotavirus vaccine impact surveillance network, Gagandeep Kang[1,6*]

Affiliations

[1] The Wellcome Trust Research Laboratory, Division of Gastrointestinal Sciences, Christian Medical College, Vellore, Tamil Nadu, India.

[2] Department of Community Health, Christian Medical College, Vellore, Tamil Nadu, India.

[3] Centers for Disease Control and Prevention, Atlanta, Georgia, USA.

[4] Indian Council of Medical Research, New Delhi, India.

[5] National Institute of Epidemiology, Chennai, India.

[6] Translational Health Science and Technology Institute, Faridabad, Haryana, India

***Corresponding author**

Gagandeep Kang

Professor,

The Wellcome Trust Research Laboratory,

Division of Gastrointestinal Sciences,

Christian Medical College, Vellore,

For peer review only

Tamil Nadu, India Pin: 632004

Telephone number: 0416 228 2052

E-mail: gkang@cmcvellore.ac.in

Word count

Abstract: 271

Main text (excluding table and references): 3669

Abstract

Introduction: Rotavirus infection accounts for 39% of under-five diarrheal deaths globally and 22% of these deaths occur in India. Introduction of rotavirus vaccine in a national immunization programme is considered to be the most effective intervention in preventing severe rotavirus disease. In 2016, India introduced an indigenous rotavirus vaccine (Rotavac®) into the Universal Immunization Programme in a phased manner. This paper describes the protocol for surveillance to monitor the performance of rotavirus vaccine following its introduction into the routine childhood immunization programme.

Methods: An active surveillance system was established to identify acute gastroenteritis cases among children less than five years of age. For all children enrolled at sentinel sites, case reporting forms are completed and a copy of vaccination record and a stool specimen obtained. The forms and specimens are sent to the referral laboratory for data entry, analysis, testing and storage. Data from sentinel sites in states that have introduced rotavirus vaccine into their routine immunization schedule will be used to determine rotavirus vaccine impact and effectiveness.

For peer review only - http://bmjopen.bmj.com/site/about/guidelines.xhtml

Ethics and dissemination: The Institutional Review Board of Christian Medical College, Vellore, and all the site institutional ethics committees approved the project. Results will be disseminated in peer-reviewed journals and with stakeholders of the universal immunization programme in India.

Discussion: This project will generate evidence on the impact and effectiveness of a rotavirus vaccine in India. Establishing a surveillance system to evaluate the impact and effectiveness of a novel rotavirus vaccine in India can serve as a guide for developing similar evaluations for vaccines against rotavirus and other vaccine preventable diseases in low- or middle-income countries.

Key words: Rotavirus, Vaccine, Impact, Surveillance, India

ARTICLE SUMMARY

Strengths and limitations of the project

- First project in India to evaluate the impact and effectiveness of a newly introduced vaccine in children
- Protocol for surveillance and monitoring
- Hospital based surveillance might not be representative of illness in the community

BACKGROUND

Diarrhea is the fourth leading cause of global under-five mortality and accounts for 9% of deaths among this age group [1]. Rotavirus infection accounts for 39% of these diarrheal deaths, the majority of which occur in low and middle income countries [2]. India accounts for 22% of the total global rotavirus mortality [3]. Rotavirus is a leading cause of moderate to severe acute

For peer review only - http://bmjopen.bmj.com/site/about/guidelines.xhtml

For peer review only

diarrhea in India; it accounts for 24% of cases of diarrhea among children less than 23 months of age and 13% of cases of diarrhea among children aged 24-59 months of age [4,5]. Introduction of rotavirus vaccine in a national immunization programme is considered to be the most effective intervention in preventing severe rotavirus disease [6,7].

The Indian National Rotavirus Surveillance Network (NRSN), established by the Indian Council of Medical Research (ICMR) in collaboration with US Centers for Disease Control and Prevention (CDC), has played an important role in documenting the disease burden of rotavirus hospitalizations in India. Established in 2005 with 7 states conducting rotavirus surveillance, the network expanded over subsequent years [8]. By 2012, 17 states and 2 union territories in India were conducting rotavirus surveillance using a standardized protocol [9]. The surveillance data highlighted the high prevalence (40%) of rotavirus in children hospitalized with diarrhea, with representation across the country [10]. Rotavirus was also found to cause significant disease burden in children <5 years of age treated as outpatients [10]. A study in Kolkata reported 48% positivity for rotavirus among outpatients over 36 months [11].

Since 2006, two live attenuated, orally-administered rotavirus vaccines have been available globally – a monovalent human rotavirus vaccine [RV1; Rotarix® (GSK Biologicals, Rixensart, Belgium)] and a pentavalent bovine-human reassortant vaccine [RV5; RotaTeq® (Merck and Co, Inc, Pennsylvania)] [12,13]. The World Health Organization (WHO) recommended inclusion of these vaccines in the routine immunization programme of all countries by 2009 [14]. Vaccination was considered cost-effective in India but concerns about affordability and long term price sustainability remained [15,16]. In March 2014, the results of the efficacy and tolerability trial of the first Indian-manufactured rotavirus vaccine (Rotavac®, Bharat Biotech) were reported [17]. Vaccine efficacy against severe rotavirus gastroenteritis in children up to 2 years of age was

For peer review only - http://bmjopen.bmj.com/site/about/guidelines.xhtml

For peer review only

55.1% (95% CI 39.9 to 66.4) for three doses of the vaccine given at 6, 10 and 14 weeks of age [18]. The National Technical Advisory Group on Immunization recommended the inclusion of Rotavac® into the universal immunization programme in 2014 based on the data from surveillance studies and the clinical trial [8,17,18]. Rotavac® is available at about Rs.64 per dose which is almost one-tenth the cost of the same vaccine in the private market and less than other rotavirus vaccines [17]. The Ministry of Health and Family Welfare accepted this recommendation, procured vaccine, and conducted training for an early phase introduction in the states of Odisha, Andhra Pradesh, Haryana and Himachal Pradesh by April 2016. In 2017, the introduction was extended to five more states, Rajasthan, Madhya Pradesh, Assam, Tripura and Tamil Nadu and will further expand to Uttar Pradesh by 2018.

The impact and effectiveness of rotavirus vaccine in other countries and regions has been studied within surveillance networks by following trends in rotavirus diarrhea hospitalizations pre- and post-vaccine introduction and/or by case-control approaches to estimate vaccine effectiveness [19–22]. Furthermore, since rotavirus accounts for a substantial proportion of all-cause and especially severe diarrhea in children, all-cause diarrheal hospitalization rates can also be used to estimate the number of hospitalizations and outpatient visits prevented by vaccine introduction.

With the introduction of an indigenous vaccine into India's Universal Immunization Programme (UIP), issues including vaccine impact on disease burden under conditions of routine use, effectiveness against currently circulating strains of rotavirus, safety of the vaccine with respect to intussusception, and cost effectiveness of the vaccination program need to be examined. Studies to examine rotavirus vaccine impact and safety using proven study designs can help answer these questions and provide support for broader introduction of rotavirus vaccine in India. The paper describes the protocol and implementation of an ongoing multisite surveillance

For peer review only - http://bmjopen.bmj.com/site/about/guidelines.xhtml

to monitor the impact and effectiveness of rotavirus vaccine following its introduction into the routine childhood immunization programme.

OBJECTIVES

The primary objectives are:

1) To identify cases of rotavirus among children less than five years hospitalized for acute gastroenteritis (AGE) and to determine the circulating rotavirus genotypes pre- and post-introduction of Rotavac® vaccine using sentinel hospital-based active surveillance

2) To measure changes in proportion of all-cause AGE and severity of presentations at the sentinel surveillance sites pre- and post-introduction of Rotavac®

3) To determine the effectiveness of a completed series of Rotavac® against laboratory-confirmed severe rotavirus AGE under conditions of routine use in India, using sentinel hospital surveillance sites and case-control methods.

The secondary objectives are:

1) To determine vaccine effectiveness against specific rotavirus genotypes,

2) To determine vaccine effectiveness of a partial series of Rotavac®,

3) To determine potential waning of Rotavac® effectiveness during the surveillance period.

METHODS/DESIGN

Project management

For peer review only - http://bmjopen.bmj.com/site/about/guidelines.xhtml

The rotavirus vaccine impact assessment project is funded by the Bill and Melinda Gates Foundation and conducted by the Christian Medical College (CMC), Vellore and the CDC, Atlanta, USA in collaboration with the ICMR, New Delhi. CMC is responsible for all administrative arrangements, while monitoring is jointly conducted by CMC and ICMR. Prior to the vaccine introduction, the Ministry of Health and Family Welfare and the ICMR wrote to the Health Secretaries in each state to inform them of the impact assessment surveillance and request their cooperation.

Site selection

The processes for sites participating in assessment of vaccine impact and effectiveness include identification and reporting of childhood diarrhea and laboratory detection of rotavirus from stool samples. When selecting sites for participation, priority was given to sites that had previously conducted surveillance under the NRSN. To identify additional sites, we selected large tertiary care hospitals in states introducing vaccine and requested their participation. A meeting was organized for potential site investigators and site representatives were requested to present retrospective data on diarrhea admissions among children under five years of age [Supplementary table 1]. The criterion for a site to participate in AGE surveillance was at least 250 under five diarrhea admissions per year. On average, of seven hospitals contacted in each state, three hospitals met these criteria and were selected [Fig.1]. For each selected participating hospital, a Memorandum of Understanding (MOU) was signed between CMC and the participating institution.

Each participating site recruited a four member team consisting of a project medical officer, a laboratory technician and two field workers. During the initiation visit, meetings were held with

For peer review only

pediatrics and community health departments where the project tools, logistics and possible timelines were discussed. The initial contact to initiation of the surveillance took at least six months for completing institutional processes and hiring and training of staff [Fig.2].

Ethical clearance

Ethical clearances were obtained from the Institutional Review Board of CMC, Vellore, and from each participating institution.

Project design and setting

This is a multi-centric surveillance project being carried out in nine states of India over a period of four years [Fig.1]. Vaccine effectiveness will be determined by a case-control evaluation. Vaccine impact will be determined by monitoring trends in rotavirus hospitalizations pre- and post-rotavirus vaccine introduction at the sites that were part of NRSN project and have baseline data [Table.1].

Table1: Participating sites in the rotavirus vaccine impact assessment surveillance

S. No.	Surveillance Network Centers	District	State	Phase
1	Kurnool Medical College	Kurnool	Andhra Pradesh	Phase I
2	Government General Hospital	Kakinada	Andhra Pradesh	Phase I
3	Andhra Medical College	Vishakhapatnam	Andhra Pradesh	Phase I
4	Sri Venkateswara Medical College*	Tirupati	Andhra Pradesh	Phase I
5	Sardar Vallabhai Patel Post Graduate Institute of Pediatrics	Cuttack	Odisha	Phase I
6	Kalinga Institute of Medical Sciences	Bhubaneshwar	Odisha	Phase I
7	Institute of Medical Sciences and SUM Hospital	Bhubaneshwar	Odisha	Phase I
8	Hi-Tech Hospital and Medical college*	Bhubaneshwar	Odisha	Phase I
9	Pandit Bhagwat Dayal Sharma Post Graduate Institute of Medical Sciences*	Rohtak	Haryana	Phase I
10	Shaheed Hasan Khan Mewati	Mewat	Haryana	Phase I

For peer review only - http://bmjopen.bmj.com/site/about/guidelines.xhtml

	Government Medical College			
11	BPS Government Medical College for Women	Sonipat	Haryana	Phase I
12	Post Graduate Institute of Medical Education and Research	Chandigarh	Chandigarh	Phase I
13	Rajendra Prasad Government Medical College*	Tanda	Himachal Pradesh	Phase I
14	Indira Gandhi Government Medical College	Shimla	Himachal Pradesh	Phase I
15	Kanchi Kama Koti Child Trust hospital	Chennai	Tamil Nadu	Phase II
16	Institute of Child health	Chennai	Tamil Nadu	Phase II
17	Government Medical College	Madurai	Tamil Nadu	Phase II
18	Christian Medical College*	Vellore	Tamil Nadu	Phase II
19	Government Vellore Medical College	Vellore	Tamil Nadu	Phase II
20	Nalam Hospital	Vellore	Tamil Nadu	Phase II
21	Narayani Hospital and Research Centre	Vellore	Tamil Nadu	Phase II
22	Sawai Man Singh Medical college	Jaipur	Rajasthan	Phase II
23	Rabindranath Tagore medical college	Udaipur	Rajasthan	Phase II
24	Dr. Sampurnanand Medical college	Jodhpur	Rajasthan	Phase II
25	Mahatma Gandhi Memorial Medical College	Indore	Madhya Pradesh	Phase II
26	Netaji Subhash Chandra Bose Medical College	Jabalpur	Madhya Pradesh	Phase II
27	Baptist Christian Hospital	Tezpur	Assam	Phase II
28	Government Medical college	Guwahati	Assam	Phase II
29	King George Medical College	Lucknow	Uttar Pradesh	Phase III
30	Institute of Medical Sciences, Banaras Hindu University	Varanasi	Uttar Pradesh	Phase III
31	BRD Medical College	Gorakhpur	Uttar Pradesh	Phase III
32	Mangala Hospital and Research Centre	Bijnor	Uttar Pradesh	Phase III

**Sites that were part of the NRSN project*

AGE case definition

AGE is defined as the occurrence of ≥ three episodes of diarrhea within a 24-hour period, for less than seven days prior to the hospital visit, not explained by an underlying medical condition. The definition of 'looser than normal stool' is based on parental perception/judgment of their child's illness and might include changes in consistency and/or volume or changing habits of the child

For peer review only - http://bmjopen.bmj.com/site/about/guidelines.xhtml

(especially among young/breast-fed infants) [23]. Severe AGE will be defined as any case with a Vesikari severity score of ≥11 [24].

Inclusion criteria

Children are eligible for enrolment in the surveillance program if they meet all of the following criteria; i) present to one of the active surveillance hospitals for treatment of AGE, ii) are <5 years of age, iii) produce a stool sample during the first 48 hours after the presentation, and iv) the child is either treated with oral or intravenous fluids in the emergency department for at least six hours or admitted to the hospital and treated with oral rehydration or intravenous fluids.

Exclusion criteria

Patients will be excluded from enrolment if any of the following apply; i) more than 5 years of age, ii) unable to obtain informed consent from the parent/caregiver or guardian, iii) admitted to another hospital for >24 hours (and subsequently transferred to the current sentinel site), iv) children from states without Rotavac® in their immunization schedule (immigrant population).

Vaccine effectiveness case-control evaluation

The vaccine effectiveness assessment against rotavirus hospitalizations will be conducted through a test-negative case-control design [25]. For the purpose of the case-control study, a child is considered vaccinated if he/she has received at least one dose of vaccine at least 14 days before gastroenteritis onset or hospital admission [17].

Cases and controls are eligible for inclusion in the vaccine effectiveness case-control evaluation if they are; i) enrolled in the active surveillance platform of a sentinel hospital, ii) age eligible to have received the vaccine, that is, at least 6 weeks of age or born after the date of vaccine

For peer review only - http://bmjopen.bmj.com/site/about/guidelines.xhtml

introduction. Cases will have a stool specimen that tests positive for rotavirus by enzyme-linked immunosorbent assay (ELISA) and controls will have a stool specimen that tests negative for rotavirus by ELISA. Cases and controls will be excluded from the vaccine effectiveness evaluation if they are enrolled prior to vaccine introduction or are age-ineligible to have received vaccine.

With regard to vaccination, efforts will be made to obtain a vaccination card, medical record or other form of documentation through which rotavirus vaccine status may be verified. Children without a vaccination record will be excluded from the vaccine effectiveness analysis.

Sample size

As trends are being monitored, continuous enrollment of children presenting to the sentinel hospitals during the evaluation period will be done. Based on recent figures, the sentinel hospitals should admit annually a combined 600-1000 children less than five years of age with AGE per state, of which approximately 200-300 children are expected to have severe rotavirus gastroenteritis.

For the vaccine effectiveness evaluation, the sample size is calculated to achieve 80% power at the 5% significance level to detect odds of 0.6 (vaccine effectiveness of 40%). The ratio of cases to controls will be 1:2. Expecting the vaccine coverage to be 80% or above for a full series of vaccinations, we would need approximately 242 cases and 484 controls to demonstrate a vaccine effectiveness of ≥40%. To enable further analyses such as genotype-specific vaccine effectiveness calculations, case recruitment will continue throughout the duration of the project irrespective of numbers achieved.

Surveillance activities

For peer review only - http://bmjopen.bmj.com/site/about/guidelines.xhtml

Per the surveillance protocol, project staff visit hospital wards daily to enroll cases admitted with acute diarrhea. Details of all inpatient diarrhea cases (0-59 months) are recorded in the diarrhea logbook. For all cases fulfilling eligibility criteria, informed consent is taken from parents/caregivers. Socio-demographic, clinical, and vaccination data are collected, along with a stool sample from the child. The interviewer reviews and photographs or photocopies the vaccination record and documents the date of administration of rotavirus vaccine, as well as other vaccines received. If the vaccination card is not available for enrolled children at the time of interview, site staff visits the child's home to review the record. If the vaccination card is not available, staff seeks more information through a visit to the local health unit where vaccinations were administered. Depending on the site, different methods including smartphones, emails, or self-addressed envelopes with stamp and money are also employed to ensure recording of accurate information.

Data flow

Stool samples and completed case report forms are sent to the referral laboratory at CMC, Vellore, monthly for testing, data entry and storage. On receipt of samples and case report forms, the shipment is checked and logged, and any issues with quantity or quality recorded. Case report forms are reviewed before data entry and data clarification forms are generated in case of any missing fields or errors and sent to sites within 3 days of receipt for clarification and responses. At the referral laboratory, stool samples are tested for rotavirus antigen using commercially available ELISA kits (Rotaclone; Meridian Biosciences, USA) and genotyped using published methods [26–28]. Rotavirus test results are reported to the sites within one month of receiving the samples and forms.

For peer review only

Monitoring surveillance

For the first three months after initiation of surveillance, sites are visited monthly. After three months, monitoring is reduced to once in every three months. At each visit, site evaluation is conducted using a monitoring checklist [Supplementary table 2], which records performance in terms of enrollment of children with diarrhea, collection of adequate stool samples and obtaining a copy of the vaccination card from all enrolled children. Every six months, a collaborators' meeting is organized to discuss the work done by each site and to enable collaborators to provide feedback to individual sites.

Analysis plan

AGE surveillance

Descriptive analyses of demographic, clinical, and treatment information will be performed to describe the children enrolled in the active surveillance program and children with specimens testing positive for rotavirus. Trends in all-cause diarrhea and rotavirus diarrhea will be assessed and compared for pre- and post-vaccine introduction using available population demographics. This will be conducted in a subset of sites that were part of the NRSN. The distributions of circulating genotypes will be described over the period and compared pre- and post-vaccine introduction. Data will be stratified by age eligibility to receive the vaccine for examining possible indirect effects among older children.

Vaccine effectiveness case-control evaluation

The primary analysis will include all verified reports of vaccine status for children who have received a full vaccine series versus no doses of the vaccine. An unconditional logistic regression

For peer review only - http://bmjopen.bmj.com/site/about/guidelines.xhtml

controlling for the date of birth and age in the model will be used. Vaccine effectiveness will be estimated using the formula ((1- odds ratio) x 100%), where the odds ratio is the adjusted odds ratio for the rotavirus immunization rate among case-patients compared with control [29,30]. If sample size permits, genotype-specific vaccine effectiveness will be calculated for the predominant circulating strains detected after vaccine introduction.

DISCUSSION

Challenges in establishing sites for impact assessment

Sites that had not previously participated in NRSN were sometimes reluctant to participate, particularly those located in remote areas. Recruitment of project staff was difficult, because of lack of suitably qualified potential staff. Staff turnover rate was high in certain hospitals, especially in rural areas. Some governmental institutions had difficulty in being able to use project funds, because their administration lacked experience in handling research projects. Recruitment and retention of project staff, particularly medical officers, is an ongoing challenge.

Challenges in project implementation

Lack of experience among site investigators in prior research resulted in the need to consistently monitor data quality. Communication with sites, especially in hilly and rural sites, is difficult, particularly with regard to telephone and email communication because of poor connectivity. These issues can be partially addressed through the use of mobile hot spots. Transport of biological specimens is an issue with courier companies, particularly because samples are shipped in temperature controlled containers.

For peer review only - http://bmjopen.bmj.com/site/about/guidelines.xhtml

For peer review only

Collection of immunization cards is crucial for the impact assessment and these cards are often not easily obtained. Therefore, multiple measures had to be implemented to ensure collection of the cards. When rotavirus vaccination was introduced, older vaccination cards available within the vaccination programme did not have space for recording the new vaccine. This required careful enquiry and verification of rotavirus vaccine receipt with the registers of Anganwadi workers [31], who are responsible for maintaining governmental immunization records for rural children.

Initially, the Rotavac® vaccine was issued as ten dose vials. Since there is no open vial policy for the vaccine, the peripheral centres withheld vaccination until they had a minimum of 7 infants before opening a new vial. In the initial period of the project, coverage was therefore low in the target age group. This was subsequently addressed by issuing five dose vials, and recommending immunization irrespective of the number of children to be immunized.

In data collection, common issues encountered include missing fields on case reporting forms, incorrect labelling of samples, inaccurate screening of hospital logs, which resulted in missing potential cases for enrolment, and errors in recording the immunization history of enrolled children. Frequent monitoring and retraining during the initial year of surveillance resulted in an improvement in performance across sites.

A limitation of this project is that rotavirus vaccination with other commercially available oral rotavirus vaccines started in the private sector, and some coverage may have been achieved before the indigenous vaccine's introduction into the country's UIP. If so, there may be delay in recruitment of cases. Additionally, herd protection has been seen in other settings [32], and if high coverage has been achieved with other rotavirus vaccines, it may be difficult to demonstrate

For peer review only - http://bmjopen.bmj.com/site/about/guidelines.xhtml

effectiveness of Rotavac®. It is also possible that low vaccination coverage in the public immunization system may result in a need to modify the analytic plan. Further, the use of test-negative controls as compared to community controls may result in biases if vaccination coverage is different among those who access the sentinel healthcare facilities and those who do not.

ETHICS AND DISSEMINATION

The project was approved by the ethics committee of CMC, Vellore and also by the ethical committees of all the participating institutes. All parents/legal guardians whose children have been hospitalized for AGE receive both oral and written information about the project. Parents/legal guardians willing to participate provide informed consent for their child's participation. Participants receive unique IDs which are used for questionnaires. The record connecting IDs with names is securely stored separate from the data, with access only for authorized personnel. All other investigators access the password protected data set without personal identifiers.

The results from the project will be communicated with all the national stakeholders, viz., Ministry of Health and Family Welfare, Govt. of India, the National Technical Advisory Group of Immunizations and the State Health Secretaries. Results will be disseminated in international and national peer-reviewed journals. The results will also be communicated at meetings and conferences.

CONCLUSIONS

This description of the protocol and challenges to extend a surveillance platform to monitor the impact and effectiveness of a newly introduced vaccine highlights the importance of robust

For peer review only - http://bmjopen.bmj.com/site/about/guidelines.xhtml

surveillance systems for vaccine preventable diseases. Such a platform is critical to document and study the performance of a new vaccine introduced into the immunization program. The rotavirus vaccine impact surveillance is expected to generate data regarding changes in proportion of all-cause AGE and severity of presentations at the sentinel surveillance sites before and after the introduction of Rotavac®, as well as permit a case-control analysis, using a test negative design. The data that we will obtain from different sites and states will help in measuring the effectiveness of the vaccine in routine programmatic use, but conduct of high quality impact assessment requires attention to process with a need to identify and apply site-specific solutions.

AUTHORS' CONTRIBUTIONS

NPN, SR, JET, MPS, UDP and GK conceived this paper. VRM, GK, SG, NPN and SR obtained all the approvals for the project. SG and GK developed and approved all the laboratory protocols. NPN wrote the first draft. All authors read, critically revised and approved the final manuscript.

FUNDING STATEMENT

This work was supported by grants from the Bill and Melinda Gates Foundation to the Centers for Disease Control and Prevention, Atlanta, GA, USA (sub-contract to CMC Vellore grant no. MOA#871-15SC) and the Translational Health Science and Technology Institute (grant no. OPP1165083).

CONSENT FOR PUBLICATION

For peer review only - http://bmjopen.bmj.com/site/about/guidelines.xhtml

Consent for publication is included in the informed consent obtained from parents/ legal guardians of all study participants.

AVAILABILITY OF DATA AND MATERIALS

The anonymized datasets generated will be shared on request.

COMPETING INTERESTS

None of the authors declared any conflict of interest.

DISCLOSURE

The findings and conclusions in this report are those of the authors and do not necessarily represent the official position of the US Centers for Disease Control and Prevention.

ACKNOWLEDGEMENTS

The authors would like to thank the investigators of all the participating institutions and the children and their families who are a part of the project. They thank Mr. J. Senthil Kumar for his help with the maps.

The investigators of rotavirus vaccine impact assessment surveillance network are Sowmiya VS, Rama Prasad GS, Goru Krishna Babu, Padmalatha Pamu, B Manohar, Subal Pradhan, Mrutunjay Dash, Nirmal Kumar Mohakud, Rajib Ray, Geetha Gathwala, Suraj Chawla, Manoj Rawal, Madhu Gupta, Sanjeev S Choudhary, Shyam Kaushik, S Balasubramaniyan, CP Girish Kumar, Sridevi A Naray an, Kulaindaivel S, Raghul Maniam, S Kumaravel, R K Gupta, Suresh Goyal, Pramod Sharma, Sharad Thora, Pawan Ghanghoriya, Koshy George, Jayanta Goswami, Ashish Wakhlu, Vineeta Gupta, Mahima Mithal, Vipin Vashishtha.

For peer review only - http://bmjopen.bmj.com/site/about/guidelines.xhtml

For peer review only

REFERENCES

1 Parashar UD, Gibson CJ, Bresee JS, *et al.* Rotavirus and severe childhood diarrhea. *Emerg Infect Dis* 2006;**12**:304–6. doi:10.3201/eid1202.050006

2 WHO | Reports. WHO. http://www.who.int/gho/publications/en/ (accessed 18 Oct 2017).

3 Tate JE, Burton AH, Boschi-Pinto C, *et al.* Global, Regional, and National Estimates of Rotavirus Mortality in Children <5 Years of Age, 2000-2013. *Clin Infect Dis Off Publ Infect Dis Soc Am* 2016;**62 Suppl 2**:S96–105. doi:10.1093/cid/civ1013

4 Kotloff KL, Nataro JP, Blackwelder WC, *et al.* Burden and aetiology of diarrhoeal disease in infants and young children in developing countries (the Global Enteric Multicenter Study, GEMS): a prospective, case-control study. *Lancet Lond Engl* 2013;**382**:209–22. doi:10.1016/S0140-6736(13)60844-2

5 Liu J, Platts-Mills JA, Juma J, *et al.* Use of quantitative molecular diagnostic methods to identify causes of diarrhoea in children: a reanalysis of the GEMS case-control study. *Lancet Lond Engl* 2016;**388**:1291–301. doi:10.1016/S0140-6736(16)31529-X

6 Esposito DH, Tate JE, Kang G, *et al.* Projected Impact and Cost-Effectiveness of a Rotavirus Vaccination Program in India, 2008. *Clin Infect Dis* 2011;**52**:171–7. doi:10.1093/cid/ciq094

7 Neogi SB, Hasan H, Sheikh K, *et al.* Scope for rotavirus vaccination in India: revisiting the scientific evidence. *Indian J Pediatr* 2011;**78**:1251–5. doi:10.1007/s12098-011-0448-3

8 Kang G, Arora R, Chitambar SD, *et al.* Multicenter, hospital-based surveillance of rotavirus disease and strains among indian children aged <5 years. *J Infect Dis* 2009;**200 Suppl 1**:S147-153. doi:10.1086/605031

9 National Rotavirus Surveillance Network, Kumar CPG, Venkatasubramanian S, *et al.* Profile and Trends of Rotavirus Gastroenteritis in Under 5 children in India, 2012 - 2014, Preliminary Report of the Indian National Rotavirus Surveillance Network. *Indian Pediatr* 2016;**53**:619–22.

10 Mullick S, Mandal P, Nayak MK, *et al.* Hospital based surveillance and genetic characterization of rotavirus strains in children (<5 years) with acute gastroenteritis in Kolkata, India, revealed resurgence of G9 and G2 genotypes during 2011-2013. *Vaccine* 2014;**32 Suppl 1**:A20-28. doi:10.1016/j.vaccine.2014.03.018

11 S. Namjoshi G, Mitra M, K. Lalwani S, *et al.* Rotavirus gastroenteritis among children less than 5 years of age in private outpatient setting in urban India. *Vaccine* 2014;**32**:A36–A44. doi:10.1016/j.vaccine.2014.03.070

12 Ruiz-Palacios GM, Pérez-Schael I, Velázquez FR, *et al.* Safety and efficacy of an attenuated vaccine against severe rotavirus gastroenteritis. *N Engl J Med* 2006;**354**:11–22. doi:10.1056/NEJMoa052434

For peer review only

For peer review only - http://bmjopen.bmj.com/site/about/guidelines.xhtml

13 Vesikari T, Matson DO, Dennehy P, *et al.* Safety and efficacy of a pentavalent human-bovine (WC3) reassortant rotavirus vaccine. *N Engl J Med* 2006;**354**:23–33. doi:10.1056/NEJMoa052664

14 Rotavirus vaccines. WHO position paper – January 2013. *Releve Epidemiol Hebd* 2013;**88**:49–64.

15 Panda S, Das A, Samanta S. Synthesizing evidences for policy translation: a public health discourse on rotavirus vaccine in India. *Vaccine* 2014;**32 Suppl 1**:A162-170. doi:10.1016/j.vaccine.2014.03.037

16 Rose J, Hawthorn RL, Watts B, *et al.* Public health impact and cost effectiveness of mass vaccination with live attenuated human rotavirus vaccine (RIX4414) in India: model based analysis. *BMJ* 2009;**339**:b3653.

17 Bhandari N, Rongsen-Chandola T, Bavdekar A, *et al.* Efficacy of a monovalent human-bovine (116E) rotavirus vaccine in Indian infants: a randomised, double-blind, placebo-controlled trial. *Lancet Lond Engl* 2014;**383**:2136–43. doi:10.1016/S0140-6736(13)62630-6

18 Mehendale S, Venkatasubramanian S, Girish Kumar CP, *et al.* Expanded Indian National Rotavirus Surveillance Network in the Context of Rotavirus Vaccine Introduction. *Indian Pediatr* 2016;**53**:575–81.

19 Ichihara MYT, Rodrigues LC, Teles Santos CAS, *et al.* Effectiveness of rotavirus vaccine against hospitalized rotavirus diarrhea: A case-control study. *Vaccine* 2014;**32**:2740–7. doi:10.1016/j.vaccine.2014.01.007

20 Velázquez RF, Linhares AC, Muñoz S, *et al.* Efficacy, safety and effectiveness of licensed rotavirus vaccines: a systematic review and meta-analysis for Latin America and the Caribbean. *BMC Pediatr* 2017;**17**. doi:10.1186/s12887-016-0771-y

21 Patel MM, Parashar UD. Assessing the effectiveness and public health impact of rotavirus vaccines after introduction in immunization programs. *J Infect Dis* 2009;**200 Suppl 1**:S291-299. doi:10.1086/605059

22 Lamberti LM, Ashraf S, Walker CLF, *et al.* A Systematic Review of the Effect of Rotavirus Vaccination on Diarrhea Outcomes Among Children Younger Than 5 Years. *Pediatr Infect Dis J* 2016;**35**:992–8. doi:10.1097/INF.0000000000001232

23 Kliegman R, Behrman RE, Nelson WE, editors. *Nelson textbook of pediatrics*. Edition 20. Phialdelphia, PA: : Elsevier 2016.

24 Ruuska T, Vesikari T. Rotavirus disease in Finnish children: use of numerical scores for clinical severity of diarrhoeal episodes. *Scand J Infect Dis* 1990;**22**:259–67. doi:10.3109/00365549009027046

25 Tate JE, Patel MM, Cortese MM, *et al.* Use of Patients With Diarrhea Who Test Negative for Rotavirus as Controls to Estimate Rotavirus Vaccine Effectiveness Through Case-Control

For peer review only - http://bmjopen.bmj.com/site/about/guidelines.xhtml

Studies. *Clin Infect Dis Off Publ Infect Dis Soc Am* 2016;**62 Suppl 2**:S106-114. doi:10.1093/cid/civ1014

26 Bines JE, Ivanoff B, Justice F, *et al.* Clinical case definition for the diagnosis of acute intussusception. *J Pediatr Gastroenterol Nutr* 2004;**39**:511–8.

27 Kang G, Desai R, Arora R, *et al.* Diversity of circulating rotavirus strains in children hospitalized with diarrhea in India, 2005-2009. *Vaccine* 2013;**31**:2879–83. doi:10.1016/j.vaccine.2013.04.030

28 Babji S, Arumugam R, Sarvanabhavan A, *et al.* Multi-center surveillance of rotavirus diarrhea in hospitalized children <5 years of age in India, 2009-2012. *Vaccine* 2014;**32 Suppl 1**:A10-12. doi:10.1016/j.vaccine.2014.03.001

29 Effectiveness of rotavirus vaccination against childhood diarrhoea in El Salvador: case-control study. - PubMed - NCBI. https://www.ncbi.nlm.nih.gov/pubmed/20551120 (accessed 6 May 2018).

30 Justino MCA, Linhares AC, Lanzieri TM, *et al.* Effectiveness of the monovalent G1P[8] human rotavirus vaccine against hospitalization for severe G2P[4] rotavirus gastroenteritis in Belém, Brazil. *Pediatr Infect Dis J* 2011;**30**:396–401. doi:10.1097/INF.0b013e3182055cc2

31 The Anganwadi Workers of India – Connecting for Health, Education, Childcare at the Grassroots Level. http://healthopine.com/the-anganwadi-workers-of-india-connecting-for-health-at-the-grassroots/ (accessed 20 Mar 2018).

32 Parashar UD, Johnson H, Steele AD, *et al.* Health Impact of Rotavirus Vaccination in Developing Countries: Progress and Way Forward. *Clin Infect Dis Off Publ Infect Dis Soc Am* 2016;**62 Suppl 2**:S91-95. doi:10.1093/cid/civ1015

Figure 1: Location of surveillance sites in states introducing Rotavac into the surveillance programme

Legends: 1 Kurnool, 2 Kakinada, 3 Vishakhapatnam, 4 Tirupati, 5 Cuttack 6, 7, 8 - Bhubaneswar, 9 Rohtak, 10 Mewat, 11 Sonipat, 12 Shimla, 13 Tanda, 14 Chandigarh, 15, 16- Chennai, 17 Madurai, 18, 19, 20, 21- Vellore, 22 Jaipur, 23 Udaipur, 24 Jodhpur, 25 Indore, 26 Jabalpur, 27 Tezpur, 28 Guwahati, 29 Lucknow, 30 Varanasi, 31 Gorakhpur, 32 Bijnor

Figure 2: Process for establishment of a new surveillance site

For peer review only - http://bmjopen.bmj.com/site/about/guidelines.xhtml

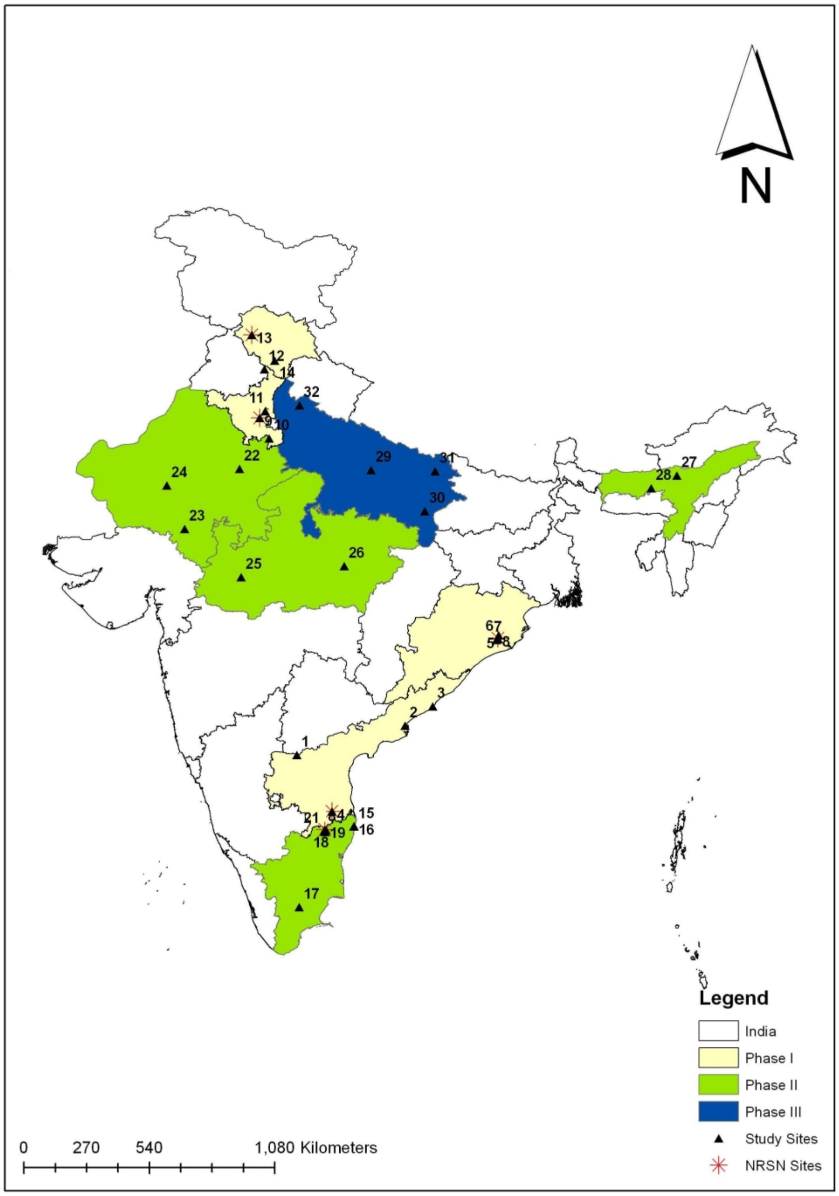

Figure1. Location of surveillance sites in states introducing Rotavac into the surveillance programme

572x803mm (96 x 96 DPI)

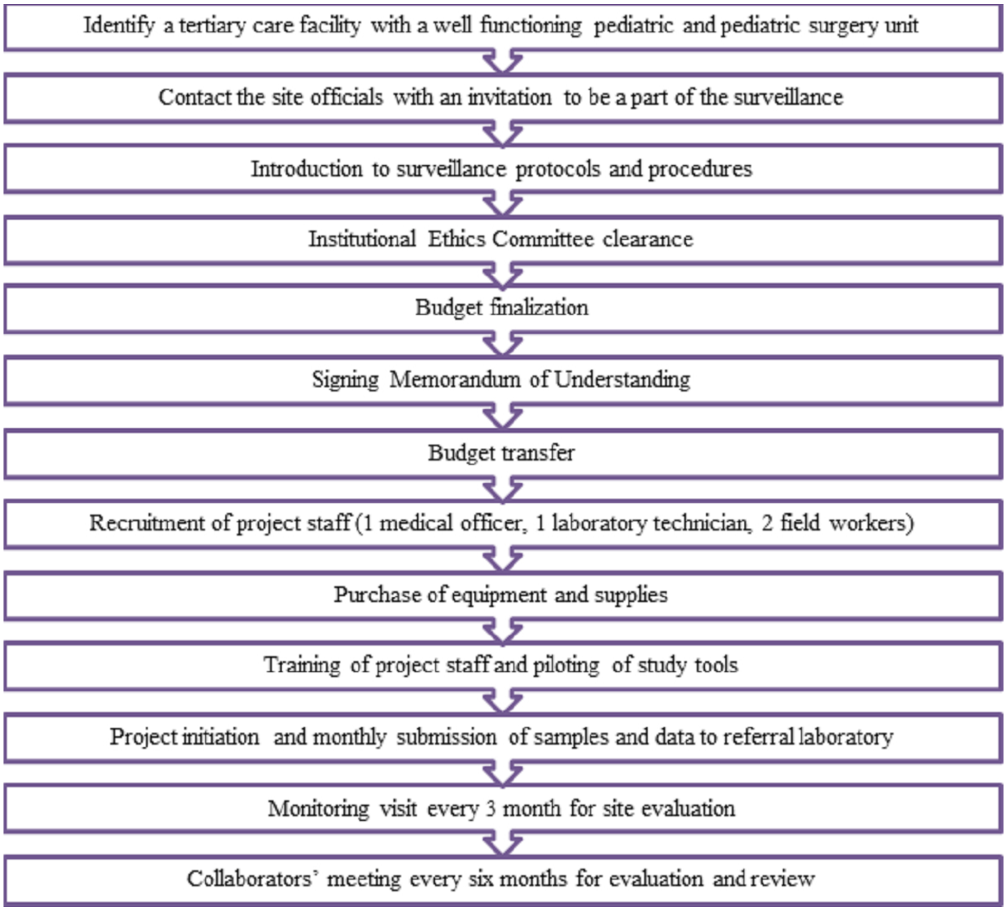

Figure2. Process for establishment of a new surveillance site

254x229mm (120 x 120 DPI)

Supplementary Table1. Baseline data from sentinel surveillance sites included in the impact assessment project

Sl. no.	Name of surveillance site	No. of under- five AGE admissions a year#
1	Kurnool Medical College, Kurnool, Andhra Pradesh	410
2	Government General hospital, Kakinada, Andhra Pradesh	400
3	King George Hospital, Vishakhapatnam, Andhra Pradesh	300
4	Sri Venkateswara Medical College, Tirupati, AP	400
5	Sardar Vallabhai Patel Post Graduate Institute of Pediatrics, Cuttack, Odisha	720
6	Kalinga Institute of Medical Sciences, Bhubaneshwar, Odisha	340
7	Institute of Medical Sciences and SUM Hospital, Bhubaneshwar, Odisha	335
8	Hi-Tech Hospital, Bhubaneshwar, Odisha	490
9	Pandit Bhagwat Dayal Sharma Post Graduate Institute of Medical Sciences, Rohtak, Haryana	250
10	Shaheed Hasan Khan Mewati Government Medical College, Mewat, Haryana	620
11	BPS Government Medical College for Women, Sonipat, Haryana	320
12	Indira Gandhi Government Medical College, Shimla, HP	200
13	Rajendra Prasad Government Medical College, Tanda, HP	300
14	Post Graduate Institute of Medical Education and Research, Chandigarh	600
15	The Institute of Child Health and Hospital for Children, Chennai, Tamil Nadu	488
16	Kanchi Kama Koti Child Trust Hospital, Chennai, Tamil Nadu	528
17	Government Medical College, Madurai, Tamil Nadu	110
18	Christian Medical College, Vellore, Tamil Nadu	
19	Nalam Hospital, Vellore, Tamil Nadu	
20	Narayani Hospital and Research Centre, Vellore, Tamil Nadu	
21	GVMC, Adukkumparai, Tamil Nadu	
22	Sawai Man Singh Medical College, Jaipur, Rajasthan	1763
23	Dr. Sampurnanand Medical College, Jodhpur,	1627

For peer review only

	Rajasthan	
24	Rabindranath Tagore Medical college, Udaipur, Rajasthan	1660
25	King George Medical College, Lucknow, Uttar Pradesh	244
26	Institute of Medical Sciences, Banaras Hindu University, Varanasi, Uttar Pradesh	144
27	BRD Medical College, Gorakhpur, Uttar Pradesh	600
28	Mangala Hospital and Research Centre, Bijnor, Uttar Pradesh	550
29	NSCB Medical College, Jabalpur, Madhya Pradesh	700
30	Mahatma Gandhi Memorial Medical college, Indore, Madhya Pradesh	710
31	Government Medical college, Guwahati, Assam	560
32	Baptist Christian Hospital, Tezpur, Assam	232

#Retrospective data for the year 2015

For peer review only

Supplementary table2: Monitoring checklist for sites doing under five diarrhea surveillance

SITE PROFILE

1. **Staff**

Name of staff
Position Title or Duties
Full-time or Part-time for rotavirus responsibilities
Percentage of time spent working on rotavirus
Years of experience in Site
Undergone training at CMC or at site

2. **Staff and equipment**

Designated place for project staff
Storage of records (ICF/CRF etc.)
Refrigerator for storage of stool samples

3. **Project management and supervision**

Lines of supervision and accountability clear to all staff
Written protocols are available and arrangements are in place for periodic review and evaluation
Arrangements are made for back-up staff to perform work during staff absences

PROJECT ACTIVITIES

1. **Recording of recruitment**

Number of children admitted for gastroenteritis
Source from which the details in the hospital logbook
How many locations are children recruited from?
Number of children enrolled for the project so far (Informed consent form completed)
Reasons for non-enrolment
Number of children for whom case report form was filled
Reasons for non-completion of CRF
Number of children for whom stool sample was collected

For peer review only

Reasons for non-collection of stool sample
Problems encountered by the site in completing this information

2. Review of informed consent forms

Number of ICFs reviewed
Location of filing the forms
Legibility
Signatures of parents/guardians
Signature of the investigator
Problems encountered by the site in completing this information

3. Completion of Case Report Forms

Number of CRFs reviewed
Number of CRFs with missing data fields
Signature and a date for completion of CRF
Record of stool sample collection
Verification of stool sample collection against the hospital logbook
Verification of stool sample collection against the sample transmittal log
Problems encountered by the site in completing this information

4. Collection of vaccination information

What are the sources of vaccination information in the CRFs
Verification of dates on the CRFs from the source
Problems encountered by the site in completing this information

5. Collection of stool samples

Collection of stool samples
Number of samples collected
Labelling of the containers
Storage of samples

For peer review only

Quantity of the collected samples
Verification of stool samples against the hospital logbook
Verification of stool samples against the CRF
Verification of stool sample collection against the sample transmittal log
Problems encountered by the site in completing this information

SUMMARY INDICATORS

Enrolment of >90% of admitted cases of gastroenteritis
Collection of adequate stools from >90% of enrolled children
Complete documentation (ICF/CRF) in >90% of enrolled children
Collection of vaccination record (patient retained/government) for >75% of enrolled children

For peer review only

STROBE Statement

Checklist of items that should be included in reports of observational studies

Section/Topic	Item No	Recommendation	Reported on Page No
**Title and abstract**	1	(*a*) Indicate the study's design with a commonly used term in the title or the abstract	1
		(*b*) Provide in the abstract an informative and balanced summary of what was done and what was found	2
**Introduction**			
Background/rationale	2	Explain the scientific background and rationale for the investigation being reported	3
Objectives	3	State specific objectives, including any prespecified hypotheses	6
**Methods**			
Study design	4	Present key elements of study design early in the paper	6
Setting	5	Describe the setting, locations, and relevant dates, including periods of recruitment, exposure, follow-up, and data collection	8
Participants	6	(*a*) *Cohort study*—Give the eligibility criteria, and the sources and methods of selection of participants. Describe methods of follow-up<br>*Case-control study*—Give the eligibility criteria, and the sources and methods of case ascertainment and control selection. Give the rationale for the choice of cases and controls<br>*Cross-sectional study*—Give the eligibility criteria, and the sources and methods of selection of participants	10
		(*b*) *Cohort study*—For matched studies, give matching criteria and number of exposed and unexposed<br>*Case-control study*—For matched studies, give matching criteria and the number of controls per case	
Variables	7	Clearly define all outcomes, exposures, predictors, potential confounders, and effect modifiers. Give diagnostic criteria, if applicable	9, 10
Data sources/measurement	8*	For each variable of interest, give sources of data and details of methods of assessment (measurement). Describe comparability of assessment methods if there is more than one group	9, 10
Bias	9	Describe any efforts to address potential sources of bias	-
Study size	10	Explain how the study size was arrived at	11
Quantitative variables	11	Explain how quantitative variables were handled in the analyses. If applicable, describe which groupings were chosen and why	13
Statistical methods	12	(*a*) Describe all statistical methods, including those used to control for confounding	-
		(*b*) Describe any methods used to examine subgroups and interactions	-
		(*c*) Explain how missing data were addressed	-
		(*d*) *Cohort study*—If applicable, explain how loss to follow-up was addressed<br>*Case-control study*—If applicable, explain how matching of cases and controls was addressed<br>*Cross-sectional study*—If applicable, describe analytical methods taking account of sampling strategy	13
		(*e*) Describe any sensitivity analyses	-

For peer review only - http://bmjopen.bmj.com/site/about/guidelines.xhtml

Section/Topic	Item No	Recommendation	Reported on Page No
**Results**			
Participants	13*	(a) Report numbers of individuals at each stage of study—eg numbers potentially eligible, examined for eligibility, confirmed eligible, included in the study, completing follow-up, and analysed	10
		(b) Give reasons for non-participation at each stage	10
		(c) Consider use of a flow diagram	-
Descriptive data	14*	(a) Give characteristics of study participants (eg demographic, clinical, social) and information on exposures and potential confounders	13
		(b) Indicate number of participants with missing data for each variable of interest	-
		(c) *Cohort study*—Summarise follow-up time (eg, average and total amount)	-
Outcome data	15*	*Cohort study*—Report numbers of outcome events or summary measures over time	-
		Case-control study—Report numbers in each exposure category, or summary measures of exposure	-
		Cross-sectional study—Report numbers of outcome events or summary measures	-
Main results	16	(*a*) Give unadjusted estimates and, if applicable, confounder-adjusted estimates and their precision (eg, 95% confidence interval). Make clear which confounders were adjusted for and why they were included	-
		(*b*) Report category boundaries when continuous variables were categorized	
		(*c*) If relevant, consider translating estimates of relative risk into absolute risk for a meaningful time period	
Other analyses	17	Report other analyses done—eg analyses of subgroups and interactions, and sensitivity analyses	-
**Discussion**			
Key results	18	Summarise key results with reference to study objectives	-
Limitations	19	Discuss limitations of the study, taking into account sources of potential bias or imprecision. Discuss both direction and magnitude of any potential bias	15
Interpretation	20	Give a cautious overall interpretation of results considering objectives, limitations, multiplicity of analyses, results from similar studies, and other relevant evidence	-
Generalisability	21	Discuss the generalisability (external validity) of the study results	16
**Other Information**			
Funding	22	Give the source of funding and the role of the funders for the present study and, if applicable, for the original study on which the present article is based	17

**Give information separately for cases and controls in case-control studies and, if applicable, for exposed and unexposed groups in cohort and cross-sectional studies.*

Note: An Explanation and Elaboration article discusses each checklist item and gives methodological background and published examples of transparent reporting. The STROBE checklist is best used in conjunction with this article (freely available on the Web sites of PLoS Medicine at http://www.plosmedicine.org/, Annals of Internal Medicine at http://www.annals.org/, and Epidemiology at http://www.epidem.com/). Information on the STROBE Initiative is available at www.strobe-statement.org.

For peer review only - http://bmjopen.bmj.com/site/about/guidelines.xhtml

Rotavirus vaccine impact assessment surveillance in India: Protocol and methods

Journal:	*BMJ Open*
Manuscript ID	bmjopen-2018-024840.R1
Article Type:	Protocol
Date Submitted by the Author:	30-Nov-2018
Complete List of Authors:	Nair, Nayana; Christian Medical College, Vellore, Department of GI Sciences<br>Reddy, Samarasimha; Christian Medical College, Vellore, Department of GI Sciences<br>Giri, Sidhartha; Christian Medical College, Vellore, Department of GI Sciences<br>Mohan, Venkata Raghava; Christian Medical College ,<br>Parashar, Umesh; Centers for Disease Control and Prevention, Atlanta<br>Tate, Jacqueline; Centers for Disease Control and Prevention<br>Shah, Minesh; Centers for Disease Control and Prevention<br>Arora, Rashmi; Indian Council of Medical Research; Translational Health Science and Technology Institute, Faridabad<br>Gupte, Mohan; Indian Council of Medical Research<br>Mehendale, Sanjay; National Institute of Epidemiology; Indian Council of Medical Research<br>Investigators of the Rotavirus vaccine impact, Surveillance Network; Christian Medical College, Wellcome Trust Research Laboratory<br>Kang, Gagandeep; Christian Medical College, Vellore, Department of GI Sciences; Translational Health Science and Technology Institute, Faridabad
**Primary Subject Heading**:	Public health
Secondary Subject Heading:	Epidemiology, Infectious diseases, Paediatrics, Research methods, Evidence based practice
Keywords:	Rotavirus, Surveillance, India, vaccine, impact

SCHOLARONE™
Manuscripts

Rotavirus vaccine impact assessment surveillance in India: Protocol and methods

Authors

Nayana P Nair[1], Samarasimha Reddy N[1], Sidhartha Giri[1], Venkata Raghava Mohan[2], Umesh D. Parashar[3], Jacqueline E. Tate[3], Minesh Pradyuman Shah[3], Rashmi Arora[4,6], Mohan D Gupte[4], Sanjay Mehendale[4,5], Investigators of the Rotavirus vaccine impact Surveillance Network, Gagandeep Kang[1,6*]

Affiliations

[1] The Wellcome Trust Research Laboratory, Division of Gastrointestinal Sciences, Christian Medical College, Vellore, Tamil Nadu, India.

[2] Department of Community Health, Christian Medical College, Vellore, Tamil Nadu, India.

[3] Centers for Disease Control and Prevention, Atlanta, Georgia, USA.

[4]Indian Council of Medical Research, New Delhi, India.

[5]National Institute of Epidemiology, Chennai, India.

[6]Translational Health Science and Technology Institute, Faridabad, Haryana, India

***Corresponding author**

Gagandeep Kang

Professor,

The Wellcome Trust Research Laboratory,

Division of Gastrointestinal Sciences,

Christian Medical College, Vellore,

For peer review only - http://bmjopen.bmj.com/site/about/guidelines.xhtml

For peer review only

Tamil Nadu, India Pin: 632004

Telephone number: 0416 228 2052

E-mail: gkang@cmcvellore.ac.in

Word count

Abstract: 214

Main text (excluding table and references): 3677

Abstract

Introduction: Rotavirus infection accounts for 39% of under-five diarrheal deaths globally and 22% of these deaths occur in India. Introduction of rotavirus vaccine in a national immunization programme is considered to be the most effective intervention in preventing severe rotavirus disease. In 2016, India introduced an indigenous rotavirus vaccine (Rotavac®) into the Universal Immunization Programme in a phased manner. This paper describes the protocol for surveillance to monitor the performance of rotavirus vaccine following its introduction into the routine childhood immunization programme.

Methods: An active surveillance system was established to identify acute gastroenteritis cases among children less than five years of age. For all children enrolled at sentinel sites, case reporting forms are completed and a copy of vaccination record and a stool specimen obtained. The forms and specimens are sent to the referral laboratory for data entry, analysis, testing and storage. Data from sentinel sites in states that have introduced rotavirus vaccine into their routine immunization schedule will be used to determine rotavirus vaccine impact and effectiveness.

For peer review only - http://bmjopen.bmj.com/site/about/guidelines.xhtml

Ethics and dissemination: The Institutional Review Board of Christian Medical College, Vellore, and all the site institutional ethics committees approved the project. Results will be disseminated in peer-reviewed journals and with stakeholders of the universal immunization programme in India.

Key words: Rotavirus, Vaccine, Impact, Surveillance, India

ARTICLE SUMMARY

Strengths and limitations of the project

- First project in India to evaluate the impact and effectiveness of a newly introduced vaccine in children
- Use of test negative control study design for effectiveness
- Hospital based surveillance might not be representative of illness or vaccine coverage in the community

BACKGROUND

Diarrhea is the fourth leading cause of global under-five mortality and accounts for 9% of deaths among this age group [1]. Rotavirus infection accounts for 39% of these diarrheal deaths, the majority of which occur in low and middle income countries [2]. India accounts for 22% of the total global rotavirus mortality [3]. As per the Global Burden of Disease Study, 21357.6 (13150.8-33967.0) deaths occur children less than five years in India due to rotavirus infection [4]. Rotavirus is a leading cause of moderate to severe acute diarrhea in India; it accounts for 24% of cases of diarrhea among children less than 23 months of age and 13% of cases of diarrhea among children aged 24-59 months of age [5,6]. An estimated 11.4 million episodes of

For peer review only - http://bmjopen.bmj.com/site/about/guidelines.xhtml

rotaviral gastroenteritis occur among under five children leading to 872,000 hospitalizations annually[7]. Introduction of rotavirus vaccine in a national immunization programme is considered to be the most effective intervention in preventing severe rotavirus disease [8,9].

The Indian National Rotavirus Surveillance Network (NRSN), established by the Indian Council of Medical Research (ICMR) in collaboration with US Centers for Disease Control and Prevention (CDC), has played an important role in documenting the disease burden of rotavirus hospitalizations in India. Established in 2005 with 7 states conducting rotavirus surveillance, the network expanded over subsequent years [10]. By 2012, 17 states and 2 union territories in India were conducting rotavirus surveillance using a standardized protocol [11]. The surveillance data highlighted the high prevalence (40%) of rotavirus in children hospitalized with diarrhea, with representation across the country [11]. Rotavirus was also found to cause significant disease burden in children <5 years of age treated for diarrhea as outpatients [12], with a study in Kolkata reporting 48% positivity for rotavirus over 36 months [13].

Since 2006, two live attenuated, orally-administered rotavirus vaccines have been available globally – a monovalent human rotavirus vaccine [RV1; Rotarix® (GSK Biologicals, Rixensart, Belgium)] and a pentavalent bovine-human reassortant vaccine [RV5; RotaTeq® (Merck and Co, Inc, Pennsylvania)] [14,15]. The World Health Organization (WHO) recommended inclusion of these vaccines in the routine immunization programme of all countries by 2009 [16]. Vaccination was considered cost-effective in India but concerns about affordability and long term price sustainability remained [17,18]. In March 2014, the results of the efficacy and tolerability trial of the first Indian-manufactured oral rotavirus vaccine (Rotavac®, Bharat Biotech) were reported [19]. Vaccine efficacy against severe rotavirus gastroenteritis in children up to 2 years of age was 55.1% (95% CI 39.9 to 66.4) for three doses of the vaccine given at 6, 10 and 14 weeks of age

For peer review only - http://bmjopen.bmj.com/site/about/guidelines.xhtml

[20]. The National Technical Advisory Group on Immunization recommended the inclusion of Rotavac® into the universal immunization programme in 2014 based on the data from surveillance studies and the clinical trial [10,19,20]. Rotavac® is available at about Rs.64 per dose which is almost one-tenth the cost of the same vaccine in the private market and less than other rotavirus vaccines [19]. The Ministry of Health and Family Welfare accepted this recommendation, procured vaccine, and conducted training for an early phase introduction in the states of Odisha, Andhra Pradesh, Haryana and Himachal Pradesh by April 2016 [21]. In 2017, the introduction was extended to five more states, Rajasthan, Madhya Pradesh, Assam, Tripura and Tamil Nadu and will further expand to Uttar Pradesh by 2018 [22]. The coverage of the third dose of rotavirus vaccine as per WHO and United Nations Children's Fund (UNICEF) estimates for 2017 in India is about 60% [23].

The impact and effectiveness of rotavirus vaccine in other countries and regions has been studied within surveillance networks by following trends in rotavirus diarrhea hospitalizations pre- and post-vaccine introduction and/or by case-control approaches to estimate vaccine effectiveness [24–27]. Furthermore, since rotavirus accounts for a substantial proportion of all-cause and especially severe diarrhea in children, all-cause diarrheal hospitalization rates can also be used to estimate the number of hospitalizations and outpatient visits prevented by vaccine introduction.

With the introduction of an indigenous vaccine into India's Universal Immunization Programme (UIP), issues including vaccine impact on disease burden under conditions of routine use, effectiveness against currently circulating strains of rotavirus, safety of the vaccine with respect to intussusception, and cost effectiveness of the vaccination program need to be examined. Studies to examine rotavirus vaccine impact and safety using proven study designs can help answer these questions and provide support for broader introduction of rotavirus vaccine in India

For peer review only - http://bmjopen.bmj.com/site/about/guidelines.xhtml

[28]. The paper describes the protocol and implementation of an ongoing multisite surveillance to monitor the impact and effectiveness of rotavirus vaccine following its introduction into the routine childhood immunization programme.

OBJECTIVES

The primary objectives are:

1) To identify cases of rotavirus among children less than five years hospitalized for acute gastroenteritis (AGE) and to determine the circulating rotavirus genotypes pre- and post-introduction of Rotavac® vaccine using sentinel hospital-based active surveillance

2) To measure changes in proportion of AGE due to all causes including rotaviral gastroenteritis and severity of presentations at the sentinel surveillance sites pre- and post-introduction of Rotavac®

3) To determine the effectiveness of a completed series of Rotavac® against laboratory-confirmed severe rotavirus AGE under conditions of routine use in India, using sentinel hospital surveillance sites and case-control methods.

The secondary objectives are:

1) To determine vaccine effectiveness against specific rotavirus genotypes,

2) To determine vaccine effectiveness of a partial series of Rotavac®,

3) To determine potential waning of Rotavac® effectiveness during the surveillance period.

METHODS/DESIGN

For peer review only - http://bmjopen.bmj.com/site/about/guidelines.xhtml

Project management

The rotavirus vaccine impact assessment project is funded by the Bill and Melinda Gates Foundation and conducted by the Christian Medical College (CMC), Vellore and the CDC, Atlanta, USA in collaboration with the ICMR, New Delhi. CMC is responsible for all administrative arrangements, while monitoring is jointly conducted by CMC and ICMR. Prior to the vaccine introduction, the Ministry of Health and Family Welfare and the ICMR wrote to the Health Secretaries in each state to inform them of the impact assessment surveillance and request their cooperation.

Site selection

The processes for sites participating in assessment of vaccine impact and effectiveness include identification and reporting of childhood diarrhea and laboratory detection of rotavirus from stool samples. When selecting sites for participation, priority was given to sites that had previously conducted surveillance under the NRSN. To identify additional sites, we selected large tertiary care hospitals in states introducing vaccine and requested their participation. A meeting was organized for potential site investigators and site representatives were requested to present retrospective data on diarrhea admissions among children under five years of age [Supplementary table 1]. The criterion for a site to participate in AGE surveillance was at least 250 under five diarrhea admissions per year. A few sites like Varanasi and Tezpur, with lower numbers were included based on their geographic location and lack of other sites. On average, of seven hospitals contacted in each state, three hospitals met these criteria and were selected [Fig.1]. For each selected participating hospital, a Memorandum of Understanding (MOU) was signed between CMC and the participating institution.

For peer review only - http://bmjopen.bmj.com/site/about/guidelines.xhtml

Each participating site recruited a four member team consisting of a project medical officer, a laboratory technician and two field workers. During the initiation visit, meetings were held with pediatrics and community health departments where the project tools, logistics and possible timelines were discussed. The initial contact to initiation of the surveillance took at least six months for completing institutional processes and hiring and training of staff [Fig.2].

Ethical clearance

Ethical clearances were obtained from the Institutional Review Board of CMC, Vellore, and from each participating institution [Supplementary Table 2].

Project design and setting

This is a multi-centric surveillance project being carried out in nine states of India over a period of four years [Fig.1]. Vaccine effectiveness will be determined by a case-control evaluation. Vaccine impact will be determined by monitoring trends in rotavirus hospitalizations pre- and post-rotavirus vaccine introduction at the five sites that have pre-introduction data [Table.1]. The other sites will have four years of surveillance after vaccine introduction.

Table1: Participating sites in the rotavirus vaccine impact assessment surveillance

S. No.	Surveillance Network Centers	District	State	Date of vaccine introduction	Phase
1	Kurnool Medical College	Kurnool	Andhra Pradesh	20/04/2016	Phase I
2	Government General Hospital	Kakinada	Andhra Pradesh	20/04/2016	Phase I
3	Andhra Medical College	Vishakhapatnam	Andhra Pradesh	20/04/2016	Phase I
4	Sri Venkateswara Medical College*	Tirupati	Andhra Pradesh	20/04/2016	Phase I
5	Sardar Vallabhai Patel Post Graduate Institute of Pediatrics	Cuttack	Odisha	26/03/2016	Phase I
6	Kalinga Institute of Medical Sciences	Bhubaneshwar	Odisha	26/03/2016	Phase I

For peer review only - http://bmjopen.bmj.com/site/about/guidelines.xhtml

7	Institute of Medical Sciences and SUM Hospital	Bhubaneshwar	Odisha	26/03/2016	Phase I
8	Hi-Tech Hospital and Medical college*	Bhubaneshwar	Odisha	26/03/2016	Phase I
9	Pandit Bhagwat Dayal Sharma Post Graduate Institute of Medical Sciences*	Rohtak	Haryana	11/04/2016	Phase I
10	Shaheed Hasan Khan Mewati Government Medical College	Mewat	Haryana	11/04/2016	Phase I
11	BPS Government Medical College for Women	Sonipat	Haryana	11/04/2016	Phase I
12	Post Graduate Institute of Medical Education and Research	Chandigarh	Chandigarh	15/03/2016	Phase I
13	Rajendra Prasad Government Medical College*	Tanda	Himachal Pradesh	05/12/2015	Phase I
14	Indira Gandhi Government Medical College	Shimla	Himachal Pradesh	11/04/2016	Phase I
15	Kanchi Kama Koti Child Trust hospital	Chennai	Tamil Nadu	20/ 09/ 2017	Phase II
16	Institute of Child health	Chennai	Tamil Nadu	20/ 09/ 2017	Phase II
17	Government Medical College	Madurai	Tamil Nadu	20/ 09/ 2017	Phase II
18	Christian Medical College*	Vellore	Tamil Nadu	20/ 09/ 2017	Phase II
19	Government Vellore Medical College	Vellore	Tamil Nadu	20/ 09/ 2017	Phase II
20	Nalam Hospital	Vellore	Tamil Nadu	20/ 09/ 2017	Phase II
21	Narayani Hospital and Research Centre	Vellore	Tamil Nadu	20/ 09/ 2017	Phase II
22	Sawai Man Singh Medical college	Jaipur	Rajasthan	23/03/2017	Phase II
23	Rabindranath Tagore medical college	Udaipur	Rajasthan	23/03/2017	Phase II
24	Dr. Sampurnanand Medical college	Jodhpur	Rajasthan	23/03/2017	Phase II
25	Mahatma Gandhi Memorial Medical College	Indore	Madhya Pradesh	02/04/2017	Phase II
26	Netaji Subhash Chandra Bose Medical College	Jabalpur	Madhya Pradesh	02/04/2017	Phase II
27	Baptist Christian Hospital	Tezpur	Assam	14/06/2017	Phase II
28	Government Medical college	Guwahati	Assam	14/06/2017	Phase II
29	King George Medical College	Lucknow	Uttar Pradesh	16/07/2018	Phase III
30	Institute of Medical Sciences, Banaras Hindu University	Varanasi	Uttar Pradesh	16/07/2018	Phase III
31	BRD Medical College	Gorakhpur	Uttar Pradesh	16/07/2018	Phase III
32	Mangala Hospital and	Bijnor	Uttar Pradesh	16/07/2018	Phase III

For peer review only - http://bmjopen.bmj.com/site/about/guidelines.xhtml

	Research Centre				

**Sites that were part of the NRSN project*

AGE case definition

AGE is defined as the occurrence of ≥ three episodes of diarrhea within a 24-hour period, for less than seven days prior to the hospital visit, not explained by an underlying medical condition. The definition of 'looser than normal stool' is based on parental perception/judgment of their child's illness and might include changes in consistency and/or volume or changing habits of the child (especially among young/breast-fed infants) [29]. Severe AGE will be defined as any case with a Vesikari severity score of ≥11 [30].

Inclusion criteria

Children are eligible for enrolment in the surveillance program if they meet all of the following criteria; i) present to one of the active surveillance hospitals for treatment of AGE, ii) are <5 years of age, iii) produce a stool sample during the first 48 hours after the presentation, and iv) the child is either treated with oral or intravenous fluids in the emergency department for at least six hours or admitted to the hospital and treated with oral rehydration or intravenous fluids.

Exclusion criteria

Patients will be excluded from enrolment if any of the following apply; i) more than 5 years of age, ii) unable to obtain informed consent from the parent/caregiver or guardian, iii) admitted to another hospital for >24 hours (and subsequently transferred to the current sentinel site), iv) children from states without Rotavac® in their immunization schedule (immigrant population).

Vaccine effectiveness case-control evaluation

For peer review only - http://bmjopen.bmj.com/site/about/guidelines.xhtml

The vaccine effectiveness assessment against rotavirus hospitalizations will be conducted through a test-negative case-control design [31]. For the purpose of the case-control study, a child is considered vaccinated if he/she has received at least one dose of vaccine at least 14 days before gastroenteritis onset or hospital admission [19].

Cases and controls are eligible for inclusion in the vaccine effectiveness case-control evaluation if they are; i) enrolled in the active surveillance platform of a sentinel hospital, ii) age eligible to have received the vaccine, that is, at least 6 weeks of age and born after the date of vaccine introduction. Hence, children aged less than 42 days at the time of vaccine introduction will be included in the vaccine effectiveness evaluation. Cases will have a stool specimen that tests positive for rotavirus by enzyme-linked immunosorbent assay (ELISA) and controls will have a stool specimen that tests negative for rotavirus by ELISA. Cases and controls will be excluded from the vaccine effectiveness evaluation if they are enrolled prior to vaccine introduction or are age-ineligible to have received vaccine.

With regard to vaccination, efforts will be made to obtain a vaccination card, medical record or other form of documentation through which rotavirus vaccine status may be verified. Children without a vaccination record will be excluded from the vaccine effectiveness analysis.

Sample size

As trends are being monitored, continuous enrollment of children presenting to the sentinel hospitals during the evaluation period will be done. Based on recent figures, the sentinel hospitals should admit annually a combined 600-1000 children less than five years of age with AGE per state, of which approximately 200-300 children are expected to have severe rotavirus gastroenteritis.

For peer review only - http://bmjopen.bmj.com/site/about/guidelines.xhtml

For the vaccine effectiveness evaluation, the sample size is calculated to achieve 80% power at the 5% significance level to detect odds ratio of 0.6 (vaccine effectiveness of 40%). The ratio of cases to controls will be 1:2. Expecting the vaccine coverage to be 80% or above for a full series of vaccinations, we would need approximately 242 cases and 484 controls to demonstrate a vaccine effectiveness of ≥40% [23,32]. To enable further analyses such as genotype-specific vaccine effectiveness calculations, case recruitment will continue throughout the duration of the project irrespective of numbers achieved.

Surveillance activities

Per the surveillance protocol, project staff visit hospital wards daily to enroll cases admitted with acute diarrhea. Details of all inpatient diarrhea cases (0-59 months) are recorded in the diarrhea logbook. For all cases fulfilling eligibility criteria, informed consent is taken from parents/caregivers. Socio-demographic, clinical, and vaccination data are collected, along with a stool sample from the child. The interviewer reviews and photographs or photocopies the vaccination record and documents the date of administration of rotavirus vaccine, as well as other vaccines received. If the vaccination card is not available for enrolled children at the time of interview, site staff visits the child's home to review the record. If the vaccination card is not available, staff seeks more information through a visit to the local health unit where vaccinations were administered. Depending on the site, different methods including smartphones, emails, or self-addressed envelopes with stamp and money are also employed to ensure recording of accurate information. We are currently able to procure 85% of vaccination cards from enrolled children.

Data flow

For peer review only - http://bmjopen.bmj.com/site/about/guidelines.xhtml

Stool samples and completed case report forms are sent to the referral laboratory at CMC, Vellore, monthly for testing, data entry and storage. On receipt of samples and case report forms, the shipment is checked and logged, and any issues with quantity or quality recorded. Case report forms are reviewed before data entry and data clarification forms are generated in case of any missing fields or errors and sent to sites within 3 days of receipt for clarification and responses. At the referral laboratory, stool samples are tested for rotavirus antigen using commercially available ELISA kits (Rotaclone; Meridian Biosciences, USA) and genotyped using published methods [33–35]. Rotavirus test results are reported to the sites within one month of receiving the samples and forms.

Monitoring surveillance

For the first three months after initiation of surveillance, sites are visited monthly. After three months, monitoring is reduced to once in every three months. At each visit, site evaluation is conducted using a monitoring checklist [Supplementary table 3], which records performance in terms of enrollment of children with diarrhea, collection of adequate stool samples and obtaining a copy of the vaccination card from all enrolled children. Every six months, a collaborators' meeting is organized to discuss the work done by each site and to enable collaborators to provide feedback to individual sites.

Analysis plan

AGE surveillance

Descriptive analyses of demographic, clinical, and treatment information will be performed to describe the children enrolled in the active surveillance program and children with specimens testing positive for rotavirus. Trends in all-cause diarrhea and rotavirus diarrhea will be

For peer review only

compared for pre- and post-vaccine introduction using available population demographics for five NRSN sites namely, SVMC, Tirupati; Hitech hospital, Bhubaneswar; PGIMS, Rohtak; RPGMC, Tanda and CMC, Vellore that have pre-vaccine data available from 2012. The distributions of circulating genotypes will be described over the period and compared pre- and post-vaccine introduction. Data will be stratified by age eligibility to receive the vaccine for examining possible indirect effects among older children.

Vaccine effectiveness case-control evaluation

The primary analysis will include all verified reports of vaccine status for children who have received a full vaccine series versus no doses of the vaccine. An unconditional logistic regression controlling for the date of birth and age in the model will be used. Vaccine effectiveness will be estimated using the formula ((1- odds ratio) x 100%), where the odds ratio is the adjusted odds ratio for the rotavirus immunization rate among case-patients compared with control [36,37]. If sample size permits, genotype-specific vaccine effectiveness will be calculated for the predominant circulating strains detected after vaccine introduction.

PATIENT AND PUBLIC INVOLVEMENT

Patients and the public were not involved in the design of the study, which is being conducted under the supervision of the government to assess vaccine impact. Data will be shared as press release and publications when available.

ETHICS AND DISSEMINATION

The project was approved by the ethics committee of CMC, Vellore and also by the ethical committees of all the participating institutes [Supplementary table. 2]. All parents/legal

For peer review only - http://bmjopen.bmj.com/site/about/guidelines.xhtml

guardians whose children have been hospitalized for AGE receive both oral and written information about the project. Parents/legal guardians willing to participate provide informed consent for their child's participation. Participants receive unique IDs which are used for questionnaires. The record connecting IDs with names is securely stored separate from the data, with access only for authorized personnel. All other investigators access the password protected data set without personal identifiers.

The results from the project will be communicated with all the national stakeholders, viz., Ministry of Health and Family Welfare, Govt. of India, the National Technical Advisory Group of Immunizations and the State Health Secretaries. Results will be disseminated in international and national peer-reviewed journals. The results will also be communicated at meetings and conferences.

DISCUSSION

Challenges in establishing sites for impact assessment

Sites that had not previously participated in NRSN were sometimes reluctant to participate, particularly those located in remote areas. Recruitment of project staff was difficult, because of lack of suitably qualified potential staff. Staff turnover rate was high in certain hospitals, especially in rural areas. Some governmental institutions had difficulty in being able to use project funds, because their administration lacked experience in handling research projects. Recruitment and retention of project staff, particularly medical officers, is an ongoing challenge.

Challenges in project implementation

For peer review only - http://bmjopen.bmj.com/site/about/guidelines.xhtml

Lack of experience among site investigators in prior research resulted in the need to consistently monitor data quality. Communication with sites, especially in hilly and rural sites, is difficult, particularly with regard to telephone and email communication because of poor connectivity. These issues can be partially addressed through the use of mobile hot spots. Transport of biological specimens is an issue with courier companies, particularly because samples are shipped in temperature controlled containers.

Collection of immunization cards is crucial for the impact assessment and these cards are often not easily obtained. Therefore, multiple measures had to be implemented to ensure collection of the cards. When rotavirus vaccination was introduced, older vaccination cards available within the vaccination programme did not have space for recording the new vaccine. This required careful enquiry and verification of rotavirus vaccine receipt with the registers of Anganwadi workers [38], who are responsible for maintaining governmental immunization records for rural children.

Initially, the Rotavac® vaccine was issued as ten dose vials. Since there is no open vial policy for the vaccine, the peripheral centres withheld vaccination until they had a minimum of 7 infants before opening a new vial. In the initial period of the project, coverage was therefore low in the target age group. This was subsequently addressed by issuing five dose vials, and recommending immunization irrespective of the number of children to be immunized.

In data collection, common issues encountered include missing fields on case reporting forms, incorrect labelling of samples, inaccurate screening of hospital logs, which resulted in missing potential cases for enrolment, and errors in recording the immunization history of enrolled

For peer review only - http://bmjopen.bmj.com/site/about/guidelines.xhtml

children. Frequent monitoring and retraining during the initial year of surveillance resulted in an improvement in performance across sites.

A limitation of this project is that rotavirus vaccination with other commercially available oral rotavirus vaccines started in the private sector, and some coverage may have been achieved before the indigenous vaccine's introduction into the country's UIP. Herd protection has been seen in other settings [39], and if high coverage has been achieved with other rotavirus vaccines, it may be difficult to demonstrate effectiveness of Rotavac®. It is also possible that low vaccination coverage in the public immunization system may result in a need to modify the analytic plan. Further, the use of test-negative controls as compared to community controls may result in biases if vaccination coverage is different among those who access the sentinel healthcare facilities and those who do not [40].

CONCLUSIONS

This description of the protocol and challenges to extend a surveillance platform to monitor the impact and effectiveness of a newly introduced vaccine highlights the importance of robust surveillance systems for vaccine preventable diseases. Such a platform is critical to document and study the performance of a new vaccine introduced into the immunization program. The rotavirus vaccine impact surveillance is expected to generate data regarding changes in proportion of AGE due to all causes including rotaviral gastroenteritis and severity of presentations at the sentinel surveillance sites before and after the introduction of Rotavac®, as well as permit a case-control analysis, using a test negative design. The data that we will obtain from different sites and states will help in measuring the effectiveness of the vaccine in routine

For peer review only - http://bmjopen.bmj.com/site/about/guidelines.xhtml

programmatic use, but conduct of high quality impact assessment requires attention to process with a need to identify and apply site-specific solutions.

AUTHORS' CONTRIBUTIONS

NPN, SR, JET, MPS, UDP, MDG, SM and GK conceived this paper. VRM, GK, RA, SG, NPN and SR obtained all the approvals for the project. SG and GK developed and approved all the laboratory protocols. NPN wrote the first draft. All authors read, critically revised and approved the final manuscript. The Investigators of Rotavirus vaccine impact, Surveillance Network are responsible for study activities and data collection from respective sentinel sites.

FUNDING STATEMENT

This work was supported by grants from the Bill and Melinda Gates Foundation to the Centers for Disease Control and Prevention, Atlanta, GA, USA (sub-contract to CMC Vellore grant no. MOA#871-15SC) and the Translational Health Science and Technology Institute (grant no. OPP1165083).

CONSENT FOR PUBLICATION

Consent for publication is included in the informed consent obtained from parents/ legal guardians of all study participants.

AVAILABILITY OF DATA AND MATERIALS

The anonymized datasets generated will be shared on request.

COMPETING INTERESTS

None of the authors declared any conflict of interest.

For peer review only - http://bmjopen.bmj.com/site/about/guidelines.xhtml

For peer review only

DISCLOSURE

The findings and conclusions in this report are those of the authors and do not necessarily represent the official position of the US Centers for Disease Control and Prevention.

ACKNOWLEDGEMENTS

The authors would like to thank the investigators of all the participating institutions and the children and their families who are a part of the project. They thank Mr. J. Senthil Kumar for his help with the maps.

The investigators of rotavirus vaccine impact assessment surveillance network are Sowmiya VS, Rama Prasad GS, Goru Krishna Babu, Padmalatha Pamu, B Manohar, Subal Pradhan, Mrutunjay Dash, Nirmal Kumar Mohakud, Rajib Ray, Geetha Gathwala, Suraj Chawla, Manoj Rawal, Madhu Gupta, Sanjeev S Choudhary, Shyam Kaushik, S Balasubramaniyan, CP Girish Kumar, Sridevi A Narayan, Kulaindaivel S, Anna Simon, R K Gupta, Suresh Goyal, Pramod Sharma, Sharad Thora, Pawan Ghanghoriya, Koshy George, Jayanta Goswami, Ashish Wakhlu, Vineeta Gupta, Mahima Mithal, Vipin Vashishtha.

REFERENCES

1 Parashar UD, Gibson CJ, Bresee JS, *et al.* Rotavirus and severe childhood diarrhea. *Emerg Infect Dis* 2006;**12**:304–6. doi:10.3201/eid1202.050006

2 World Health Organization. *World health statistics 2018: monitoring health for the SDGs*. 2018. http://apps.who.int/iris/bitstream/handle/10665/272596/9789241565585-eng.pdf?ua=1 (accessed 29 Nov 2018).

3 Tate JE, Burton AH, Boschi-Pinto C, *et al.* Global, Regional, and National Estimates of Rotavirus Mortality in Children <5 Years of Age, 2000-2013. *Clin Infect Dis Off Publ Infect Dis Soc Am* 2016;**62 Suppl 2**:S96–105. doi:10.1093/cid/civ1013

4 Troeger C, Forouzanfar M, Rao PC, *et al.* Estimates of global, regional, and national morbidity, mortality, and aetiologies of diarrhoeal diseases: a systematic analysis for the

For peer review only - http://bmjopen.bmj.com/site/about/guidelines.xhtml

Global Burden of Disease Study 2015. *Lancet Infect Dis* 2017;**17**:909–48. doi:10.1016/S1473-3099(17)30276-1

5 Kotloff KL, Nataro JP, Blackwelder WC, *et al.* Burden and aetiology of diarrhoeal disease in infants and young children in developing countries (the Global Enteric Multicenter Study, GEMS): a prospective, case-control study. *Lancet Lond Engl* 2013;**382**:209–22. doi:10.1016/S0140-6736(13)60844-2

6 Liu J, Platts-Mills JA, Juma J, *et al.* Use of quantitative molecular diagnostic methods to identify causes of diarrhoea in children: a reanalysis of the GEMS case-control study. *Lancet Lond Engl* 2016;**388**:1291–301. doi:10.1016/S0140-6736(16)31529-X

7 John J, Sarkar R, Muliyil J, *et al.* Rotavirus gastroenteritis in India, 2011–2013: Revised estimates of disease burden and potential impact of vaccines. *Vaccine* 2014;**32**:A5–9. doi:10.1016/j.vaccine.2014.03.004

8 Esposito DH, Tate JE, Kang G, *et al.* Projected Impact and Cost-Effectiveness of a Rotavirus Vaccination Program in India, 2008. *Clin Infect Dis* 2011;**52**:171–7. doi:10.1093/cid/ciq094

9 Neogi SB, Hasan H, Sheikh K, *et al.* Scope for rotavirus vaccination in India: revisiting the scientific evidence. *Indian J Pediatr* 2011;**78**:1251–5. doi:10.1007/s12098-011-0448-3

10 Kang G, Arora R, Chitambar SD, *et al.* Multicenter, hospital-based surveillance of rotavirus disease and strains among indian children aged <5 years. *J Infect Dis* 2009;**200 Suppl 1**:S147-153. doi:10.1086/605031

11 National Rotavirus Surveillance Network, Kumar CPG, Venkatasubramanian S, *et al.* Profile and Trends of Rotavirus Gastroenteritis in Under 5 children in India, 2012 - 2014, Preliminary Report of the Indian National Rotavirus Surveillance Network. *Indian Pediatr* 2016;**53**:619–22.

12 Mullick S, Mandal P, Nayak MK, *et al.* Hospital based surveillance and genetic characterization of rotavirus strains in children (<5 years) with acute gastroenteritis in Kolkata, India, revealed resurgence of G9 and G2 genotypes during 2011-2013. *Vaccine* 2014;**32 Suppl 1**:A20-28. doi:10.1016/j.vaccine.2014.03.018

13 S. Namjoshi G, Mitra M, K. Lalwani S, *et al.* Rotavirus gastroenteritis among children less than 5 years of age in private outpatient setting in urban India. *Vaccine* 2014;**32**:A36–A44. doi:10.1016/j.vaccine.2014.03.070

14 Ruiz-Palacios GM, Pérez-Schael I, Velázquez FR, *et al.* Safety and efficacy of an attenuated vaccine against severe rotavirus gastroenteritis. *N Engl J Med* 2006;**354**:11–22. doi:10.1056/NEJMoa052434

15 Vesikari T, Matson DO, Dennehy P, *et al.* Safety and efficacy of a pentavalent human-bovine (WC3) reassortant rotavirus vaccine. *N Engl J Med* 2006;**354**:23–33. doi:10.1056/NEJMoa052664

For peer review only - http://bmjopen.bmj.com/site/about/guidelines.xhtml

16 Rotavirus vaccines. WHO position paper – January 2013. *Releve Epidemiol Hebd* 2013;**88**:49–64.

17 Panda S, Das A, Samanta S. Synthesizing evidences for policy translation: a public health discourse on rotavirus vaccine in India. *Vaccine* 2014;**32 Suppl 1**:A162-170. doi:10.1016/j.vaccine.2014.03.037

18 Rose J, Hawthorn RL, Watts B, *et al.* Public health impact and cost effectiveness of mass vaccination with live attenuated human rotavirus vaccine (RIX4414) in India: model based analysis. *BMJ* 2009;**339**:b3653.

19 Bhandari N, Rongsen-Chandola T, Bavdekar A, *et al.* Efficacy of a monovalent human-bovine (116E) rotavirus vaccine in Indian infants: a randomised, double-blind, placebo-controlled trial. *Lancet Lond Engl* 2014;**383**:2136–43. doi:10.1016/S0140-6736(13)62630-6

20 Mehendale S, Venkatasubramanian S, Girish Kumar CP, *et al.* Expanded Indian National Rotavirus Surveillance Network in the Context of Rotavirus Vaccine Introduction. *Indian Pediatr* 2016;**53**:575–81.

21 Shri J P Nadda launches Rotavirus vaccine as part of Universal Immunization Programme; terms it a "historic moment." http://pib.nic.in/newsite/PrintRelease.aspx?relid=138342 (accessed 29 Nov 2018).

22 Shri JP Nadda launchesExpansion of Rotavirus vaccine under Universal Immunization Programme. http://pib.nic.in/newsite/PrintRelease.aspx?relid=158549 (accessed 29 Nov 2018).

23 India: WHO & UNICEF estimates of immunization coverage: 2017 revision. http://www.who.int/immunization/monitoring_surveillance/data/ind.pdf (accessed 25 Oct 2018).

24 Ichihara MYT, Rodrigues LC, Teles Santos CAS, *et al.* Effectiveness of rotavirus vaccine against hospitalized rotavirus diarrhea: A case-control study. *Vaccine* 2014;**32**:2740–7. doi:10.1016/j.vaccine.2014.01.007

25 Velázquez RF, Linhares AC, Muñoz S, *et al.* Efficacy, safety and effectiveness of licensed rotavirus vaccines: a systematic review and meta-analysis for Latin America and the Caribbean. *BMC Pediatr* 2017;**17**. doi:10.1186/s12887-016-0771-y

26 Patel MM, Parashar UD. Assessing the effectiveness and public health impact of rotavirus vaccines after introduction in immunization programs. *J Infect Dis* 2009;**200 Suppl 1**:S291-299. doi:10.1086/605059

27 Lamberti LM, Ashraf S, Walker CLF, *et al.* A Systematic Review of the Effect of Rotavirus Vaccination on Diarrhea Outcomes Among Children Younger Than 5 Years. *Pediatr Infect Dis J* 2016;**35**:992–8. doi:10.1097/INF.0000000000001232

For peer review only - http://bmjopen.bmj.com/site/about/guidelines.xhtml

28 Reddy S, Nair NP, Giri S, *et al.* Safety monitoring of ROTAVAC vaccine and etiological investigation of intussusception in India: study protocol. *BMC Public Health* 2018;**18**:898. doi:10.1186/s12889-018-5809-7

29 Kliegman R, Behrman RE, Nelson WE, editors. *Nelson textbook of pediatrics*. Edition 20. Phialdelphia, PA: : Elsevier 2016.

30 Ruuska T, Vesikari T. Rotavirus disease in Finnish children: use of numerical scores for clinical severity of diarrhoeal episodes. *Scand J Infect Dis* 1990;**22**:259–67. doi:10.3109/00365549009027046

31 Tate JE, Patel MM, Cortese MM, *et al.* Use of Patients With Diarrhea Who Test Negative for Rotavirus as Controls to Estimate Rotavirus Vaccine Effectiveness Through Case-Control Studies. *Clin Infect Dis Off Publ Infect Dis Soc Am* 2016;**62 Suppl 2**:S106-114. doi:10.1093/cid/civ1014

32 Government of India. Ministry of Health and Family Welfare. Mission Indradhanush. http://pib.nic.in/newsite/PrintRelease.aspx?relid=181816 (accessed 25 Oct 2018).

33 Bines JE, Ivanoff B, Justice F, *et al.* Clinical case definition for the diagnosis of acute intussusception. *J Pediatr Gastroenterol Nutr* 2004;**39**:511–8.

34 Kang G, Desai R, Arora R, *et al.* Diversity of circulating rotavirus strains in children hospitalized with diarrhea in India, 2005-2009. *Vaccine* 2013;**31**:2879–83. doi:10.1016/j.vaccine.2013.04.030

35 Babji S, Arumugam R, Sarvanabhavan A, *et al.* Multi-center surveillance of rotavirus diarrhea in hospitalized children <5 years of age in India, 2009-2012. *Vaccine* 2014;**32 Suppl 1**:A10-12. doi:10.1016/j.vaccine.2014.03.001

36 Palma O de, Cruz L, Ramos H, *et al.* Effectiveness of rotavirus vaccination against childhood diarrhoea in El Salvador: case-control study. undefined. 2010./paper/Effectiveness-of-rotavirus-vaccination-against-in-Palma-Cruz/a4eac970bf37d83d9d841d4b8351415ed2f86bad (accessed 25 Oct 2018).

37 Justino MCA, Linhares AC, Lanzieri TM, *et al.* Effectiveness of the monovalent G1P[8] human rotavirus vaccine against hospitalization for severe G2P[4] rotavirus gastroenteritis in Belém, Brazil. *Pediatr Infect Dis J* 2011;**30**:396–401. doi:10.1097/INF.0b013e3182055cc2

38 The Anganwadi Workers of India – Connecting for Health, Education, Childcare at the Grassroots Level. http://healthopine.com/the-anganwadi-workers-of-india-connecting-for-health-at-the-grassroots/ (accessed 20 Mar 2018).

39 Parashar UD, Johnson H, Steele AD, *et al.* Health Impact of Rotavirus Vaccination in Developing Countries: Progress and Way Forward. *Clin Infect Dis Off Publ Infect Dis Soc Am* 2016;**62 Suppl 2**:S91-95. doi:10.1093/cid/civ1015

For peer review only

For peer review only - http://bmjopen.bmj.com/site/about/guidelines.xhtml

40 Haber M, Lopman BA, Tate JE, *et al.* A comparison of the test-negative and traditional case-control study designs with respect to the bias of estimates of rotavirus vaccine effectiveness. *Vaccine* 2018;**36**:5071–6. doi:10.1016/j.vaccine.2018.06.072

Figure 1: Location of surveillance sites in states introducing Rotavac into the universal immunization programme

Legends: 1 Kurnool, 2 Kakinada, 3 Vishakhapatnam, 4 Tirupati, 5 Cuttack 6, 7, 8 - Bhubaneswar, 9 Rohtak, 10 Mewat, 11 Sonipat, 12 Shimla, 13 Tanda, 14 Chandigarh, 15, 16- Chennai, 17 Madurai, 18, 19, 20, 21- Vellore, 22 Jaipur, 23 Udaipur, 24 Jodhpur, 25 Indore, 26 Jabalpur, 27 Tezpur, 28 Guwahati, 29 Lucknow, 30 Varanasi, 31 Gorakhpur, 32 Bijnor

Figure 2: Process for establishment of a new surveillance site

For peer review only

For peer review only - http://bmjopen.bmj.com/site/about/guidelines.xhtml

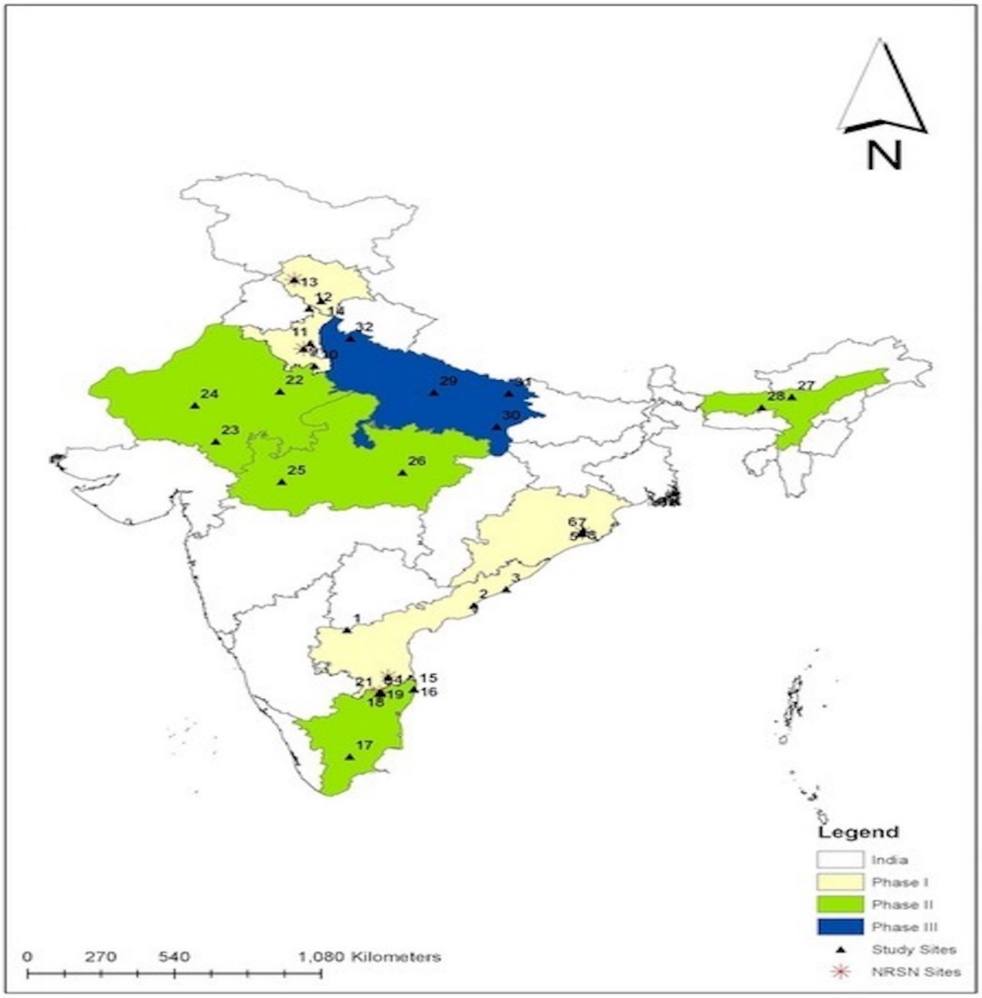

Figure 1: Location of surveillance sites in states introducing Rotavac into the Universal Immunization Programme

90x90mm (300 x 300 DPI)

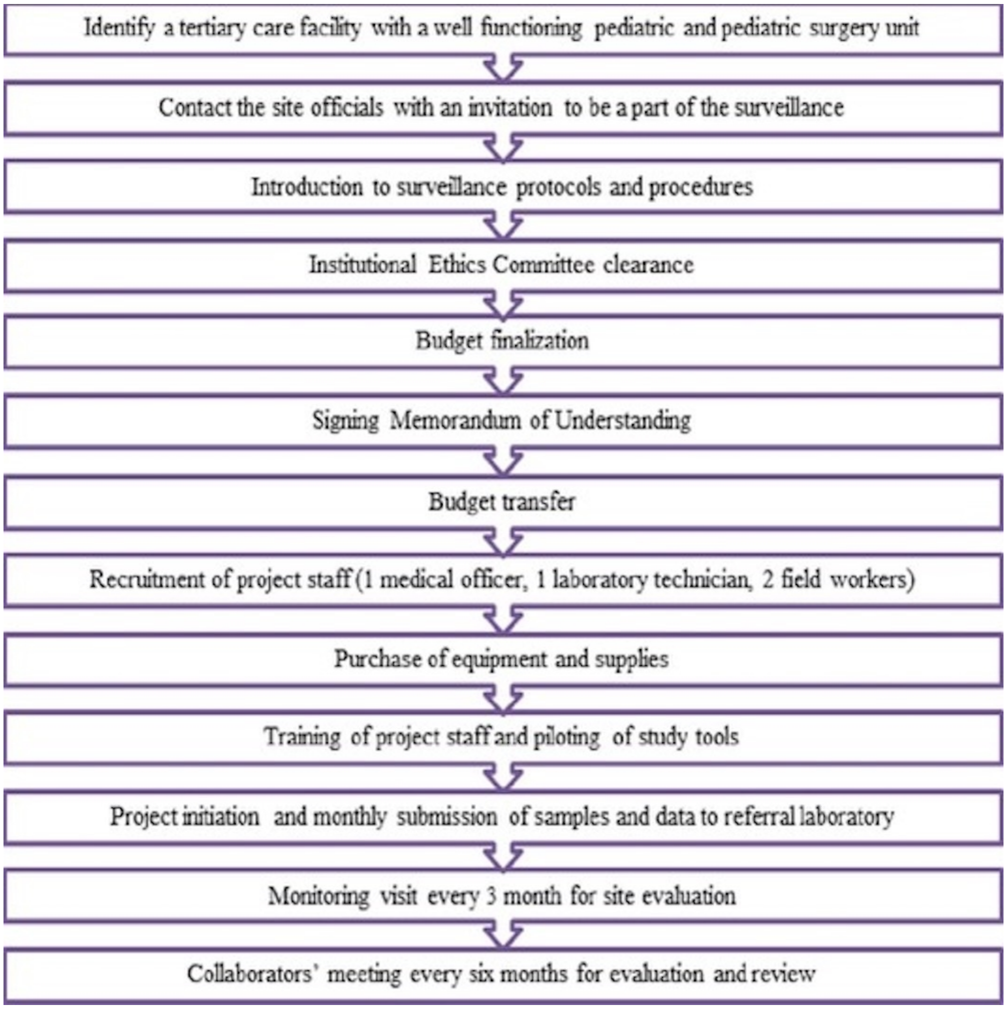

Figure 2: Process of establishment of a new surveillance site

90x90mm (300 x 300 DPI)

SupplementaryTable1. Baseline data from sentinel surveillance sites included in the impact assessment project

Sl. no.	Name of surveillance site	No. of under- five AGE admissions a year#
1	Kurnool Medical College, Kurnool, Andhra Pradesh	410
2	Government General hospital, Kakinada, Andhra Pradesh	400
3	King George Hospital, Vishakhapatnam, Andhra Pradesh	300
4	Sri Venkateswara Medical College, Tirupati, AP	400
5	Sardar Vallabhai Patel Post Graduate Institute of Pediatrics, Cuttack, Odisha	720
6	Kalinga Institute of Medical Sciences, Bhubaneshwar, Odisha	340
7	Institute of Medical Sciences and SUM Hospital, Bhubaneshwar, Odisha	335
8	Hi-Tech Hospital, Bhubaneshwar, Odisha	490
9	Pandit Bhagwat Dayal Sharma Post Graduate Institute of Medical Sciences, Rohtak, Haryana	250
10	Shaheed Hasan Khan Mewati Government Medical College, Mewat, Haryana	620
11	BPS Government Medical College for Women, Sonipat, Haryana	320
12	Indira Gandhi Government Medical College, Shimla, HP	200
13	Rajendra Prasad Government Medical College, Tanda, HP	300
14	Post Graduate Institute of Medical Education and Research, Chandigarh	600

For peer review only

15	The Institute of Child Health and Hospital for Children, Chennai, Tamil Nadu	488
16	Kanchi Kama Koti Child Trust Hospital, Chennai, Tamil Nadu	528
17	Government Medical College, Madurai, Tamil Nadu	110
18	Christian Medical College, Vellore, Tamil Nadu	
19	Nalam Hospital, Vellore, Tamil Nadu	
20	Narayani Hospital and Research Centre, Vellore, Tamil Nadu	
21	GVMC, Adukkumparai, Tamil Nadu	
22	Sawai Man Singh Medical College, Jaipur, Rajasthan	1763
23	Dr. Sampurnanand Medical College, Jodhpur, Rajasthan	1627
24	Rabindranath Tagore Medical college, Udaipur, Rajasthan	1660
25	King George Medical College, Lucknow, Uttar Pradesh	244
26	Institute of Medical Sciences, Banaras Hindu University, Varanasi, Uttar Pradesh	144
27	BRD Medical College, Gorakhpur, Uttar Pradesh	600
28	Mangala Hospital and Research Centre, Bijnor, Uttar Pradesh	550
29	NSCB Medical College, Jabalpur, Madhya Pradesh	700
30	Mahatma Gandhi Memorial Medical college, Indore, Madhya Pradesh	710
31	Government Medical college, Guwahati, Assam	560
32	Baptist Christian Hospital, Tezpur, Assam	232

#Retrospective data for the year 2015

For peer review only

Supplementary Table 2: Ethical committee details for Protocol submission

Sl. No.	Name of the institution	Name of the ethical committee (Reference number)
1	Kurnool Medical College, Kurnool, Andhra Pradesh	Institutional Ethical committee of Kurnool Medical College, Kurnool dated 12.11.2015
2	Government General Hospital, Kakinada, Andhra Pradesh	Institutional Ethics Committee cell of Rangaraya Medical College (IEC/RMC/2017/241 dated 06.05.2017)
3	Andhra Medical College, Vishakhapatnam, Andhra Pradesh	Institutional Ethical committee of King George Hospital, Vishakhapatnam (proposal 1 dated 19.11.2016)
4	Sri Venkateswara Medical College, Tirupati, Andhra Pradesh	Institutional Ethical committee of S.V Medical College (Lr. No. 2/2016 dated 07.02.2016)
5	Sardar Vallabhai Patel Post Graduate Institute of Pediatrics, Cuttack, Odisha	Institutional Ethical committee of SCB Medical College, Cuttack (IEC no. 273 dated 4.4.2016)
6	Kalinga Institute of Medical Sciences, Bhubaneswar, Odisha	Ethics committee of Kalinga Institute of Medical Sciences, Bhubaneswar (KIMS/Dir/11/16 dated 6.01.2016)
7	Institute of Medical Sciences and SUM Hospital, Bhubaneswar, Odisha	Institutional Ethical Committee of Institute of Medical Sciences and SUM Hospital, Bhubaneswar (Letter no. 232/20/11/2015 dated 20.11.2015)
8	Hi-Tech Hospital and Medical college, Bhubaneswar, Odisha	Institutional Ethical Committee of Hi-Tech Medical College and Hospital (HMCH/IEC/15/3067 dated 29.12.2015)
9	Pandit Bhagwat Dayal Sharma Post Graduate Institute of Medical Sciences, Rohtak, Haryana	Institutional Ethics Committee, Pandit Bhagwat Dayal Sharma Post Graduate Institute of

For peer review only

		Medical Sciences, Rohtak (No.IEC/16/41 dated 28.03.2016)
10	Shaheed Hasan Khan Mewati Government Medical College, Mewat, Haryana	Institutional Ethics Committee, Shaheed Hasan Khan Mewati Government Medical College, Mewat (SHKM/CM/2016/890 dated 13.02.2016)
11	BPS Government Medical College for Women, Sonipat, Haryana	Institutional Ethics Committee, BPS Government Medical College for Women, Sonipat (No. Peds/BPSGMC/16 dated 01.04.2016)
12	Post Graduate Institute of Medical Education and Research, Chandigarh	Institutional Ethics Committee, Post Graduate Institute of Medical Education and Research, Chandigarh (PGI/IEC/2016/3267 dated 03.08.2016)
13	Rajendra Prasad Government Medical College, Tanda, Himachal Pradesh	Institutional Ethics Committee, Rajendra Prasad Government Medical College, Tanda (HFW-H-DRPGMC/Ethics/2016 dated 21.01.2016)
14	Indira Gandhi Government Medical College, Shimla, Himachal Pradesh	Institutional Ethics Committee, Indira Gandhi Government Medical College, Shimla (ECR/533/Inst/HP/2014 dated 13.05.2016)
15	Kanchi Kama Koti Child Trust hospital, Chennai, Tamil Nadu	Ethics committee of Kanchi Kama Koti Childs Trust Hospital and the Childs Trust (CTMRF/EC/S/02/2017 dated 18.03.2017)
16	Institute of Child health, Egmore, Tamil Nadu	Institutional Ethics Committee of Madras Medical College, Chennai (No. 01052017 dated 02.05.2017)

For peer review only

17	Government Medical College, Madurai, Tamil Nadu	Ethics Committee of Madurai Medical College, Madurai (dated 27.11.2017)
18	Christian Medical College, Vellore, Tamil Nadu	Institutional Review Board, Christian Medical College, Vellore (IRB Min. no. 9530 dated 22.07.2015)
19	Government Vellore Medical College, Vellore, Tamil Nadu	Institutional Review Board, Christian Medical College, Vellore (IRB Min. no. 9530 dated 22.07.2015)
20	Nalam Hospital, Vellore, Tamil Nadu	Institutional Review Board, Christian Medical College, Vellore (IRB Min. no. 9530 dated 22.07.2015)
21	Narayani Hospital and Research Centre, Vellore, Tamil Nadu	Institutional Review Board, Christian Medical College, Vellore (IRB Min. no. 9530 dated 22.07.2015)
22	Sawai Man Singh Medical college, Jaipur, Rajasthan	Office of the Ethics Committee, Sawai Man Singh Medical college, Jaipur (3205/MC/EC/2017 dated 08.06.2017)
23	Rabindranath Tagore Medical College, Udaipur, Rajasthan	Ethics Committee, Rabindranath Tagore Medical College, Udaipur (RNT/STAT/IEC/2017/54 dated 18.04.2017)
24	Dr. Sampurnanand Medical College, Jodhpur, Rajasthan	Institutional Ethics Committee, Dr. Sampurnanand Medical College, Jodhpur (No.F.1/Acad/MC/JU/17/16277 dated 20.06.2017)
25	Mahatma Gandhi Memorial Medical College, Indore, Madhya Pradesh	Institutional Ethics Committee, Mahatma Gandhi Memorial

For peer review only

		Medical College, Indore (dated 24.05.2017)
26	Netaji Subhash Chandra Bose Medical College, Jabalpur, Madhya Pradesh	Institutional Review Board, Netaji Subhash Chandra Bose Medical College, Jabalpur, Madhya Pradesh
27	Baptist Christian Hospital, Tezpur, Assam	Institutional Ethics Committee, Emmanuel Hospital Association, New Delhi (Protocol No. 163 dated 23.03.2018)
28	Government Medical college, Guwahati, Assam	Institutional Review Board, Government Medical college, Guwahati (dated 28.02.2018)
29	King George Medical College, Lucknow, Uttar Pradesh	Institutional Ethics Committee, King George Medical University, Lucknow (No.515/Ethics/R-cell-17 dated 26.05.2017)
30	Institute of Medical Sciences, Banaras Hindu University, Varanasi, Uttar Pradesh	Institutional Ethics Committee, Institute of Medical Sciences, Banaras Hindu University, Varanasi (Dean/2016-17/EC/734 dated 31.03.2017)
31	Baba Ram Das Medical College, Gorakhpur, Uttar Pradesh	Institutional Review Board, Baba Ram Das Medical College, Gorakhpur, Uttar Pradesh (dated 20.07.2018)
32	Mangala Hospital and Research Centre, Bijnor, Uttar Pradesh	Institutional Ethics Committee, Mangala Hospital and Research Centre, Bijnor (IEC/MH/2018/01 dated 24.01.2018)

For peer review only

Supplementary table 3: Monitoring checklist for sites doing under five diarrhea surveillance

SITE PROFILE

1. Staff

Name of staff
Position Title or Duties
Full-time or Part-time for rotavirus responsibilities
Percentage of time spent working on rotavirus
Years of experience in Site
Undergone training at CMC or at site

2. Staff and equipment

Designated place for project staff
Storage of records (ICF/CRF etc.)
Refrigerator for storage of stool samples

3. Project management and supervision

Lines of supervision and accountability clear to all staff
Written protocols are available, and arrangements are in place for periodic review and evaluation
Arrangements are made for back-up staff to perform work during staff absences

PROJECT ACTIVITIES

1. Recording of recruitment

Number of children admitted for gastroenteritis
Source from which the details in the hospital logbook like ward registers, online information, emergency records etc.
How many locations are children recruited from?

For peer review only

Number of children enrolled for the project so far (Informed consent form completed)
Reasons for non-enrolment
Number of children for whom case report form was filled
Reasons for non-completion of CRF
Number of children for whom stool sample was collected
Reasons for non-collection of stool sample
Problems encountered by the site in completing this information

2. Review of informed consent forms

Number of ICFs reviewed
Location of filing the forms
Legibility of the consent forms especially name, date and signature
Signatures of parents/guardians
Signature of the investigator
Problems encountered by the site in completing this information

3. Completion of Case Report Forms

Number of CRFs reviewed
Number of CRFs with missing data fields
Signature and a date for completion of CRF
Record of stool sample collection
Verification of stool sample collection against the hospital logbook
Verification of stool sample collection against the sample transmittal log

For peer review only

Problems encountered by the site in completing this information

4. Collection of vaccination information

What are the sources of vaccination information in the CRFs
Verification of dates on the CRFs from the source
Problems encountered by the site in completing this information

5. Collection of stool samples

Collection of stool samples
Number of samples collected
Labelling of the containers
Storage of samples in the deep freezer including temperature maintenance and positioning of the samples
Quantity of the collected samples
Verification of stool samples against the hospital logbook
Verification of stool samples against the CRF
Verification of stool sample collection against the sample transmittal log
Problems encountered by the site in completing this information

SUMMARY INDICATORS

Enrolment of >90% of admitted cases of gastroenteritis
Collection of adequate stools from >90% of enrolled children
Complete documentation (ICF/CRF) in >90% of enrolled children
Collection of vaccination record (patient retained/government) for >75% of enrolled children

For peer review only

BMJ Open

Rotavirus vaccine impact assessment surveillance in India: Protocol and methods

Journal:	*BMJ Open*
Manuscript ID	bmjopen-2018-024840.R2
Article Type:	Protocol
Date Submitted by the Author:	22-Feb-2019
Complete List of Authors:	Nair, Nayana; Christian Medical College, Vellore, Department of GI Sciences<br>Reddy, Samarasimha; Christian Medical College, Vellore, Department of GI Sciences<br>Giri, Sidhartha; Christian Medical College, Vellore, Department of GI Sciences<br>Mohan, Venkata Raghava; Christian Medical College ,<br>Parashar, Umesh; Centers for Disease Control and Prevention, Atlanta<br>Tate, Jacqueline; Centers for Disease Control and Prevention<br>Shah, Minesh; Centers for Disease Control and Prevention<br>Arora, Rashmi; Indian Council of Medical Research; Translational Health Science and Technology Institute, Faridabad<br>Gupte, Mohan; Indian Council of Medical Research<br>Mehendale, Sanjay; National Institute of Epidemiology; Indian Council of Medical Research<br>Investigators of the Rotavirus vaccine impact, Surveillance Network; Christian Medical College, Wellcome Trust Research Laboratory<br>Kang, Gagandeep; Christian Medical College, Vellore, Department of GI Sciences; Translational Health Science and Technology Institute, Faridabad
**Primary Subject Heading**:	Public health
Secondary Subject Heading:	Epidemiology, Infectious diseases, Paediatrics, Research methods, Evidence based practice
Keywords:	Rotavirus, Surveillance, India, vaccine, impact

SCHOLARONE™
Manuscripts

Rotavirus vaccine impact assessment surveillance in India: Protocol and methods

Authors

Nayana P Nair[1], Samarasimha Reddy N[1], Sidhartha Giri[1], Venkata Raghava Mohan[2], Umesh D. Parashar[3], Jacqueline E. Tate[3], Minesh Pradyuman Shah[3], Rashmi Arora[4,6], Mohan D Gupte[4], Sanjay Mehendale[4,5], Investigators of the Rotavirus vaccine impact Surveillance Network, Gagandeep Kang[1,6*]

Affiliations

[1] The Wellcome Trust Research Laboratory, Division of Gastrointestinal Sciences, Christian Medical College, Vellore, Tamil Nadu, India.

[2] Department of Community Health, Christian Medical College, Vellore, Tamil Nadu, India.

[3] Centers for Disease Control and Prevention, Atlanta, Georgia, USA.

[4]Indian Council of Medical Research, New Delhi, India.

[5]National Institute of Epidemiology, Chennai, India.

[6]Translational Health Science and Technology Institute, Faridabad, Haryana, India

***Corresponding author**

Gagandeep Kang

Professor,

The Wellcome Trust Research Laboratory,

Division of Gastrointestinal Sciences,

Christian Medical College, Vellore,

For peer review only - http://bmjopen.bmj.com/site/about/guidelines.xhtml

Tamil Nadu, India Pin: 632004

Telephone number: 0416 228 2052

E-mail: gkang@cmcvellore.ac.in

Word count

Abstract: 214

Main text (excluding table and references): 3764

Abstract

Introduction: Rotavirus infection accounts for 39% of under-five diarrheal deaths globally and 22% of these deaths occur in India. Introduction of rotavirus vaccine in a national immunization programme is considered to be the most effective intervention in preventing severe rotavirus disease. In 2016, India introduced an indigenous rotavirus vaccine (Rotavac®) into the Universal Immunization Programme in a phased manner. This paper describes the protocol for surveillance to monitor the performance of rotavirus vaccine following its introduction into the routine childhood immunization programme.

Methods: An active surveillance system was established to identify acute gastroenteritis cases among children less than five years of age. For all children enrolled at sentinel sites, case reporting forms are completed and a copy of vaccination record and a stool specimen obtained. The forms and specimens are sent to the referral laboratory for data entry, analysis, testing and storage. Data from sentinel sites in states that have introduced rotavirus vaccine into their routine immunization schedule will be used to determine rotavirus vaccine impact and effectiveness.

For peer review only - http://bmjopen.bmj.com/site/about/guidelines.xhtml

Ethics and dissemination: The Institutional Review Board of Christian Medical College, Vellore, and all the site institutional ethics committees approved the project. Results will be disseminated in peer-reviewed journals and with stakeholders of the universal immunization programme in India.

Key words: Rotavirus, Vaccine, Impact, Surveillance, India

ARTICLE SUMMARY

Strengths and limitations of the project

- First project in India to evaluate the impact and effectiveness of a newly introduced vaccine in children
- Use of test negative control design, a convenient and low cost technique used in effectiveness studies for several vaccines, including rotavirus vaccines
- Hospital based surveillance might not be representative of illness or vaccine coverage in the community

BACKGROUND

Diarrhea is the fourth leading cause of global under-five mortality and accounts for 9% of deaths among this age group [1]. Rotavirus infection accounts for 39% of these diarrheal deaths, the majority of which occur in low and middle income countries [2]. India accounts for 22% of the total global rotavirus mortality [3]. As per the Global Burden of Disease Study, 21357.6 (13150.8-33967.0) deaths occur children less than five years in India due to rotavirus infection [4]. Rotavirus is a leading cause of moderate to severe acute diarrhea in India; it accounts for 24% of cases of diarrhea among children less than 23 months of age and 13% of cases of

For peer review only - http://bmjopen.bmj.com/site/about/guidelines.xhtml

diarrhea among children aged 24-59 months of age [5,6]. An estimated 11.4 million episodes of rotaviral gastroenteritis occur among under five children leading to 872,000 hospitalizations annually[7]. Introduction of rotavirus vaccine in a national immunization programme is considered to be the most effective intervention in preventing severe rotavirus disease [8,9].

The Indian National Rotavirus Surveillance Network, established by the Indian Council of Medical Research (ICMR) in collaboration with US Centers for Disease Control and Prevention (CDC), has played an important role in documenting the disease burden of rotavirus hospitalizations in India. Established in 2005 with 7 states conducting rotavirus surveillance, the network expanded over subsequent years [10]. By 2012, 17 states and 2 union territories in India were conducting rotavirus surveillance using a standardized protocol [11]. The surveillance data highlighted the high prevalence (40%) of rotavirus in children hospitalized with diarrhea, with representation across the country [11]. Rotavirus was also found to cause significant disease burden in children <5 years of age treated for diarrhea as outpatients [12], with a study in Kolkata reporting 48% positivity for rotavirus over 36 months [13].

Since 2006, two live attenuated, orally-administered rotavirus vaccines have been available globally – a monovalent human rotavirus vaccine [RV1; Rotarix® (GSK Biologicals, Rixensart, Belgium)] and a pentavalent bovine-human reassortant vaccine [RV5; RotaTeq® (Merck and Co, Inc, Pennsylvania)] [14,15]. The World Health Organization (WHO) recommended inclusion of these vaccines in the routine immunization programme of all countries by 2009 [16]. Vaccination was considered cost-effective in India but concerns about affordability and long term price sustainability remained [17,18]. In March 2014, the results of the efficacy and tolerability trial of the first Indian-manufactured oral rotavirus vaccine (Rotavac®, Bharat Biotech) were reported [19]. Vaccine efficacy against severe rotavirus gastroenteritis in children up to 2 years of age was

For peer review only - http://bmjopen.bmj.com/site/about/guidelines.xhtml

For peer review only

55.1% (95% CI 39.9 to 66.4) for three doses of the vaccine given at 6, 10 and 14 weeks of age [20]. The National Technical Advisory Group on Immunization recommended the inclusion of Rotavac® into the universal immunization programme in 2014 based on the data from surveillance studies and the clinical trial [10,19,20]. Rotavac® is available at about Rs.64 per dose which is almost one-tenth the cost of the same vaccine in the private market and less than other rotavirus vaccines [19]. The Ministry of Health and Family Welfare accepted this recommendation, procured vaccine, and conducted training for an early phase introduction in the states of Odisha, Andhra Pradesh, Haryana and Himachal Pradesh by April 2016 [21]. In 2017, the introduction was extended to five more states, Rajasthan, Madhya Pradesh, Assam, Tripura and Tamil Nadu and will further expand to Uttar Pradesh by 2018 [22]. The coverage of the third dose of rotavirus vaccine as per WHO and United Nations Children's Fund (UNICEF) estimates for 2017 in India is about 60% [23].

The impact and effectiveness of rotavirus vaccine in other countries and regions has been studied within surveillance networks by following trends in rotavirus diarrhea hospitalizations pre- and post-vaccine introduction and/or by case-control approaches to estimate vaccine effectiveness [24–27]. Furthermore, since rotavirus accounts for a substantial proportion of all-cause and especially severe diarrhea in children, all-cause diarrheal hospitalization rates can also be used to estimate the number of hospitalizations and outpatient visits prevented by vaccine introduction.

With the introduction of an indigenous vaccine into India's Universal Immunization Programme (UIP), issues including vaccine impact on disease burden under conditions of routine use, effectiveness against currently circulating strains of rotavirus, safety of the vaccine with respect to intussusception, and cost effectiveness of the vaccination program need to be examined. Studies to examine rotavirus vaccine impact and safety using proven study designs can help

For peer review only - http://bmjopen.bmj.com/site/about/guidelines.xhtml

answer these questions and provide support for broader introduction of rotavirus vaccine in India [28]. The paper describes the protocol and implementation of an ongoing multisite surveillance to monitor the impact and effectiveness of rotavirus vaccine following its introduction into the routine childhood immunization programme.

OBJECTIVES

The primary objectives are:

1) To identify cases of rotavirus among children less than five years hospitalized for acute gastroenteritis (AGE) and to determine the circulating rotavirus genotypes pre- and post-introduction of Rotavac® vaccine using sentinel hospital-based active surveillance

2) To measure changes in proportion of AGE due to all causes including rotaviral gastroenteritis and severity of presentations at the sentinel surveillance sites pre- and post-introduction of Rotavac®

3) To determine the effectiveness of a completed series of Rotavac® against laboratory-confirmed severe rotavirus AGE under conditions of routine use in India, using sentinel hospital surveillance sites and case-control methods.

The secondary objectives are:

1) To determine vaccine effectiveness against specific rotavirus genotypes,

2) To determine vaccine effectiveness of a partial series of Rotavac®,

3) To determine potential waning of Rotavac® effectiveness during the surveillance period.

METHODS/DESIGN

For peer review only - http://bmjopen.bmj.com/site/about/guidelines.xhtml

For peer review only

Project management

The rotavirus vaccine impact assessment project is funded by the Bill and Melinda Gates Foundation and conducted by the Christian Medical College (CMC), Vellore and the CDC, Atlanta, USA in collaboration with the ICMR, New Delhi. CMC is responsible for all administrative arrangements, while monitoring is jointly conducted by CMC and ICMR. Prior to the vaccine introduction, the Ministry of Health and Family Welfare and the ICMR wrote to the Health Secretaries in each state to inform them of the impact assessment surveillance and request their cooperation.

Site selection

The processes for sites participating in assessment of vaccine impact and effectiveness include identification and reporting of childhood diarrhea and laboratory detection of rotavirus from stool samples. When selecting sites for participation, priority was given to sites that had previously conducted surveillance under the National Rota Surveillance Network (NRSN). To identify additional sites, we selected large tertiary care hospitals in states introducing vaccine and requested their participation. A meeting was organized for potential site investigators and site representatives were requested to present retrospective data on diarrhea admissions among children under five years of age [Supplementary table 1]. The criterion for a site to participate in AGE surveillance was at least 250 under five diarrhea admissions per year. A few sites like Varanasi and Tezpur, with lower numbers were included based on their geographic location and lack of other sites. On average, of seven hospitals contacted in each state, three hospitals met these criteria and were selected [Fig.1]. For each selected participating hospital, a Memorandum of Understanding (MOU) was signed between CMC and the participating institution.

For peer review only - http://bmjopen.bmj.com/site/about/guidelines.xhtml

Each participating site recruited a four member team consisting of a project medical officer, a laboratory technician and two field workers. During the initiation visit, meetings were held with pediatrics and community health departments where the project tools, logistics and possible timelines were discussed. The initial contact to initiation of the surveillance took at least six months for completing institutional processes and hiring and training of staff [Fig.2].

Ethical clearance

Ethical clearances were obtained from the Institutional Review Board of CMC, Vellore, and from each participating institution [Supplementary Table 2].

Project design and setting

This is a multi-centric surveillance project being carried out in nine states of India over a period of four years [Fig.1]. Vaccine effectiveness will be determined by a case-control evaluation. Vaccine impact will be determined by monitoring trends in rotavirus hospitalizations pre- and post-rotavirus vaccine introduction at the five sites that have pre-introduction data [Table.1]. The other sites will have four years of surveillance after vaccine introduction.

Table1: Participating sites in the rotavirus vaccine impact assessment surveillance

S. No.	Surveillance Network Centers	District	State	Date of vaccine introduction	Phase
1	Kurnool Medical College	Kurnool	Andhra Pradesh	20/04/2016	Phase I
2	Government General Hospital	Kakinada	Andhra Pradesh	20/04/2016	Phase I
3	Andhra Medical College	Vishakhapatnam	Andhra Pradesh	20/04/2016	Phase I
4	Sri Venkateswara Medical College*	Tirupati	Andhra Pradesh	20/04/2016	Phase I

For peer review only - http://bmjopen.bmj.com/site/about/guidelines.xhtml

5	Sardar Vallabhai Patel Post Graduate Institute of Pediatrics	Cuttack	Odisha	26/03/2016	Phase I
6	Kalinga Institute of Medical Sciences	Bhubaneshwar	Odisha	26/03/2016	Phase I
7	Institute of Medical Sciences and SUM Hospital	Bhubaneshwar	Odisha	26/03/2016	Phase I
8	Hi-Tech Hospital and Medical college*	Bhubaneshwar	Odisha	26/03/2016	Phase I
9	Pandit Bhagwat Dayal Sharma Post Graduate Institute of Medical Sciences*	Rohtak	Haryana	11/04/2016	Phase I
10	Shaheed Hasan Khan Mewati Government Medical College	Mewat	Haryana	11/04/2016	Phase I
11	BPS Government Medical College for Women	Sonipat	Haryana	11/04/2016	Phase I
12	Post Graduate Institute of Medical Education and Research	Chandigarh	Chandigarh	15/03/2016	Phase I
13	Rajendra Prasad Government Medical College*	Tanda	Himachal Pradesh	05/12/2015	Phase I
14	Indira Gandhi Government Medical College	Shimla	Himachal Pradesh	11/04/2016	Phase I
15	Kanchi Kama Koti Child Trust hospital	Chennai	Tamil Nadu	20/ 09/ 2017	Phase II
16	Institute of Child health	Chennai	Tamil Nadu	20/ 09/ 2017	Phase II
17	Government Medical College	Madurai	Tamil Nadu	20/ 09/ 2017	Phase II
18	Christian Medical College*	Vellore	Tamil Nadu	20/ 09/ 2017	Phase II
19	Government Vellore Medical College	Vellore	Tamil Nadu	20/ 09/ 2017	Phase II

For peer review only

For peer review only - http://bmjopen.bmj.com/site/about/guidelines.xhtml

20	Nalam Hospital	Vellore	Tamil Nadu	20/ 09/ 2017	Phase II
21	Narayani Hospital and Research Centre	Vellore	Tamil Nadu	20/ 09/ 2017	Phase II
22	Sawai Man Singh Medical college	Jaipur	Rajasthan	23/03/2017	Phase II
23	Rabindranath Tagore medical college	Udaipur	Rajasthan	23/03/2017	Phase II
24	Dr. Sampurnanand Medical college	Jodhpur	Rajasthan	23/03/2017	Phase II
25	Mahatma Gandhi Memorial Medical College	Indore	Madhya Pradesh	02/04/2017	Phase II
26	Netaji Subhash Chandra Bose Medical College	Jabalpur	Madhya Pradesh	02/04/2017	Phase II
27	Baptist Christian Hospital	Tezpur	Assam	14/06/2017	Phase II
28	Government Medical college	Guwahati	Assam	14/06/2017	Phase II
29	King George Medical College	Lucknow	Uttar Pradesh	16/07/2018	Phase III
30	Institute of Medical Sciences, Banaras Hindu University	Varanasi	Uttar Pradesh	16/07/2018	Phase III
31	BRD Medical College	Gorakhpur	Uttar Pradesh	16/07/2018	Phase III
32	Mangala Hospital and Research Centre	Bijnor	Uttar Pradesh	16/07/2018	Phase III

**Sites that were part of the NRSN project*

AGE case definition

AGE is defined as the occurrence of ≥ three episodes of diarrhea within a 24-hour period, for less than seven days prior to the hospital visit, not explained by an underlying medical condition. The definition of 'looser than normal stool' is based on parental perception/judgment of their child's illness and might include changes in consistency and/or volume or changing habits of the child

For peer review only - http://bmjopen.bmj.com/site/about/guidelines.xhtml

(especially among young/breast-fed infants) [29]. Severe AGE will be defined as any case with a Vesikari severity score of ≥11 [30].

Inclusion criteria

Children are eligible for enrolment in the surveillance program if they meet all of the following criteria; i) present to one of the active surveillance hospitals for treatment of AGE, ii) are <5 years of age, iii) produce a stool sample during the first 48 hours after the presentation, and iv) the child is either treated with oral or intravenous fluids in the emergency department for at least six hours or admitted to the hospital and treated with oral rehydration or intravenous fluids.

Exclusion criteria

Patients will be excluded from enrolment if any of the following apply; i) more than 5 years of age, ii) unable to obtain informed consent from the parent/caregiver or guardian, iii) admitted to another hospital for >24 hours (and subsequently transferred to the current sentinel site), iv) children from states without Rotavac® in their immunization schedule (immigrant population).

Vaccine effectiveness case-control evaluation

The vaccine effectiveness assessment against rotavirus hospitalizations will be conducted through a test-negative case-control design [31]. The test negative study design is a convenient and low cost method for estimation of effectiveness of a vaccine as the study can be facility- rather than community-based and since commercially available enzyme-linked immunosorbent assay (ELISA) kits have a sensitivity and specificity of over 99% [32], the probability of any bias is negligible [33]. For the purpose of the case-control study, a child is considered vaccinated if

For peer review only

For peer review only - http://bmjopen.bmj.com/site/about/guidelines.xhtml

he/she has received at least one dose of vaccine at least 14 days before gastroenteritis onset or hospital admission [19].

Cases and controls are eligible for inclusion in the vaccine effectiveness case-control evaluation if they are; i) enrolled in the active surveillance platform of a sentinel hospital, ii) age eligible to have received the vaccine, that is, at least 6 weeks of age and born after the date of vaccine introduction. Hence, children aged less than 42 days at the time of vaccine introduction will be included in the vaccine effectiveness evaluation. Cases will have a stool specimen that tests positive for rotavirus by ELISA and controls will have a stool specimen that tests negative for rotavirus by ELISA. Cases and controls will be excluded from the vaccine effectiveness evaluation if they are enrolled prior to vaccine introduction or are age-ineligible to have received vaccine.

With regard to vaccination, efforts will be made to obtain a vaccination card, medical record or other form of documentation through which rotavirus vaccine status may be verified. Children without a vaccination record will be excluded from the vaccine effectiveness analysis.

Sample size

As trends are being monitored, continuous enrollment of children presenting to the sentinel hospitals during the evaluation period will be done. Based on recent figures, the sentinel hospitals should admit annually a combined 600-1000 children less than five years of age with AGE per state, of which approximately 200-300 children are expected to have severe rotavirus gastroenteritis.

For the vaccine effectiveness evaluation, the sample size is calculated to achieve 80% power at the 5% significance level to detect odds ratio of 0.6 (vaccine effectiveness of 40%). The ratio of

For peer review only - http://bmjopen.bmj.com/site/about/guidelines.xhtml

cases to controls will be 1:2. Expecting the vaccine coverage to be 80% or above for a full series of vaccinations, we would need approximately 242 cases and 484 controls to demonstrate a vaccine effectiveness of ≥40% [23,34]. To enable further analyses such as genotype-specific vaccine effectiveness calculations, case recruitment will continue throughout the duration of the project irrespective of numbers achieved.

Surveillance activities

Per the surveillance protocol, project staff visit hospital wards daily to enroll cases admitted with acute diarrhea. Details of all inpatient diarrhea cases (0-59 months) are recorded in the diarrhea logbook. For all cases fulfilling eligibility criteria, informed consent is taken from parents/caregivers. Socio-demographic, clinical, and vaccination data are collected, along with a stool sample from the child. The interviewer reviews and photographs or photocopies the vaccination record and documents the date of administration of rotavirus vaccine, as well as other vaccines received. If the vaccination card is not available for enrolled children at the time of interview, site staff visits the child's home to review the record. If the vaccination card is not available, staff seeks more information through a visit to the local health unit where vaccinations were administered. Depending on the site, different methods including smartphones, emails, or self-addressed envelopes with stamp and money are also employed to ensure recording of accurate information. We are currently able to procure 85% of vaccination cards from enrolled children.

Data flow

Stool samples and completed case report forms are sent to the referral laboratory at CMC, Vellore, monthly for testing, data entry and storage. On receipt of samples and case report forms,

For peer review only - http://bmjopen.bmj.com/site/about/guidelines.xhtml

the shipment is checked and logged, and any issues with quantity or quality recorded. Case report forms are reviewed before data entry and data clarification forms are generated in case of any missing fields or errors and sent to sites within 3 days of receipt for clarification and responses. At the referral laboratory, stool samples are tested for rotavirus antigen using commercially available ELISA kits (Rotaclone; Meridian Biosciences, USA) and genotyped using published methods [35–37]. Rotavirus test results are reported to the sites within one month of receiving the samples and forms.

Monitoring surveillance

For the first three months after initiation of surveillance, sites are visited monthly. After three months, monitoring is reduced to once in every three months. At each visit, site evaluation is conducted using a monitoring checklist [Supplementary table 3], which records performance in terms of enrollment of children with diarrhea, collection of adequate stool samples and obtaining a copy of the vaccination card from all enrolled children. Every six months, a collaborators' meeting is organized to discuss the work done by each site and to enable collaborators to provide feedback to individual sites.

Analysis plan

AGE surveillance

Descriptive analyses of demographic, clinical, and treatment information will be performed to describe the children enrolled in the active surveillance program and children with specimens testing positive for rotavirus. Trends in all-cause diarrhea will be compared for pre- and post-vaccine introduction using available population demographics for five NRSN sites, Tirupati (SVMC); Bhubaneswar (Hitech hospital); Rohtak (PGIMS); Tanda (RPGMC) and Vellore

For peer review only - http://bmjopen.bmj.com/site/about/guidelines.xhtml

(CMC) that have hospital based pre-vaccine data on AGE among under five children from 2012 onwards. For these 5 sites, a simple comparison of rotavirus diarrhea before and after the vaccine introduction will be estimated. The distributions of circulating genotypes will be described over the period and compared pre- and post-vaccine introduction. Data will be stratified by age eligibility to receive the vaccine for examining possible indirect effects among older children.

Vaccine effectiveness case-control evaluation

The primary analysis will include all verified reports of vaccine status for children who have received a full vaccine series versus no doses of the vaccine. An unconditional logistic regression controlling for the date of birth and age in the model will be used. Vaccine effectiveness will be estimated using the formula ((1- odds ratio) x 100%), where the odds ratio is the adjusted odds ratio for the rotavirus immunization rate among case-patients compared with control [38,39]. If sample size permits, genotype-specific vaccine effectiveness will be calculated for the predominant circulating strains detected after vaccine introduction.

PATIENT AND PUBLIC INVOLVEMENT

Patients and the public were not involved in the design of the study, which is being conducted under the supervision of the government to assess vaccine impact. Data will be shared as press release and publications when available.

ETHICS AND DISSEMINATION

The project was approved by the ethics committee of CMC, Vellore and also by the ethical committees of all the participating institutes [Supplementary table. 2]. All parents/legal guardians whose children have been hospitalized for AGE receive both oral and written

For peer review only - http://bmjopen.bmj.com/site/about/guidelines.xhtml

information about the project. Parents/legal guardians willing to participate provide informed consent for their child's participation. Participants receive unique IDs which are used for questionnaires. The record connecting IDs with names is securely stored separate from the data, with access only for authorized personnel. All other investigators access the password protected data set without personal identifiers.

The results from the project will be communicated with all the national stakeholders, viz., Ministry of Health and Family Welfare, Govt. of India, the National Technical Advisory Group of Immunizations and the State Health Secretaries. Results will be disseminated in international and national peer-reviewed journals. The results will also be communicated at meetings and conferences.

DISCUSSION

Challenges in establishing sites for impact assessment

Sites that had not previously participated in NRSN were sometimes reluctant to participate, particularly those located in remote areas. Recruitment of project staff was difficult, because of lack of suitably qualified potential staff. Staff turnover rate was high in certain hospitals, especially in rural areas. Some governmental institutions had difficulty in being able to use project funds, because their administration lacked experience in handling research projects. Recruitment and retention of project staff, particularly medical officers, is an ongoing challenge.

Challenges in project implementation

Lack of experience among site investigators in prior research resulted in the need to consistently monitor data quality. Communication with sites, especially in hilly and rural sites, is difficult,

For peer review only - http://bmjopen.bmj.com/site/about/guidelines.xhtml

particularly with regard to telephone and email communication because of poor connectivity. These issues can be partially addressed through the use of mobile hot spots. Transport of biological specimens is an issue with courier companies, particularly because samples are shipped in temperature controlled containers.

Collection of immunization cards is crucial for the impact assessment and these cards are often not easily obtained. Therefore, multiple measures had to be implemented to ensure collection of the cards. When rotavirus vaccination was introduced, older vaccination cards available within the vaccination programme did not have space for recording the new vaccine. This required careful enquiry and verification of rotavirus vaccine receipt with the registers of Anganwadi workers [40], who are responsible for maintaining governmental immunization records for rural children.

Initially, the Rotavac® vaccine was issued as ten dose vials. Since there is no open vial policy for the vaccine, the peripheral centres withheld vaccination until they had a minimum of 7 infants before opening a new vial. In the initial period of the project, coverage was therefore low in the target age group. This was subsequently addressed by issuing five dose vials, and recommending immunization irrespective of the number of children to be immunized.

In data collection, common issues encountered include missing fields on case reporting forms, incorrect labelling of samples, inaccurate screening of hospital logs, which resulted in missing potential cases for enrolment, and errors in recording the immunization history of enrolled children. Frequent monitoring and retraining during the initial year of surveillance resulted in an improvement in performance across sites.

For peer review only - http://bmjopen.bmj.com/site/about/guidelines.xhtml

A limitation of this project is that rotavirus vaccination with other commercially available oral rotavirus vaccines started in the private sector, and some coverage may have been achieved before the indigenous vaccine's introduction into the country's UIP. Herd protection has been seen in other settings [41], and if high coverage has been achieved with other rotavirus vaccines, it may be difficult to demonstrate effectiveness of Rotavac®. It is also possible that low vaccination coverage in the public immunization system may result in a need to modify the analytic plan. Further, the use of test-negative controls as compared to community controls may result in minimal bias if vaccination coverage is different among those who access the sentinel healthcare facilities and those who do not [33].

CONCLUSIONS

This description of the protocol and challenges to extend a surveillance platform to monitor the impact and effectiveness of a newly introduced vaccine highlights the importance of robust surveillance systems for vaccine preventable diseases. Such a platform is critical to document and study the performance of a new vaccine introduced into the immunization program. The rotavirus vaccine impact surveillance is expected to generate data regarding changes in proportion of AGE due to all causes including rotaviral gastroenteritis and severity of presentations at the sentinel surveillance sites before and after the introduction of Rotavac®, as well as permit a case-control analysis, using a test negative design. The data that we will obtain from different sites and states will help in measuring the effectiveness of the vaccine in routine programmatic use, but conduct of high quality impact assessment requires attention to process with a need to identify and apply site-specific solutions.

AUTHORS' CONTRIBUTIONS

For peer review only - http://bmjopen.bmj.com/site/about/guidelines.xhtml

NPN, SR, JET, MPS, UDP, MDG, SM and GK conceived this paper. VRM, GK, RA, SG, NPN and SR obtained all the approvals for the project. SG and GK developed and approved all the laboratory protocols. NPN wrote the first draft. All authors read, critically revised and approved the final manuscript. The Investigators of Rotavirus vaccine impact, Surveillance Network are responsible for study activities and data collection from respective sentinel sites.

FUNDING STATEMENT

This work was supported by grants from the Bill and Melinda Gates Foundation to the Centers for Disease Control and Prevention, Atlanta, GA, USA (sub-contract to CMC Vellore grant no. MOA#871-15SC) and the Translational Health Science and Technology Institute (grant no. OPP1165083).

CONSENT FOR PUBLICATION

Consent for publication is included in the informed consent obtained from parents/ legal guardians of all study participants.

AVAILABILITY OF DATA AND MATERIALS

The anonymized datasets generated will be shared on request.

COMPETING INTERESTS

None of the authors declared any conflict of interest.

DISCLOSURE

The findings and conclusions in this report are those of the authors and do not necessarily represent the official position of the US Centers for Disease Control and Prevention.

For peer review only - http://bmjopen.bmj.com/site/about/guidelines.xhtml

For peer review only

ACKNOWLEDGEMENTS

The authors would like to thank the investigators of all the participating institutions and the children and their families who are a part of the project. They thank Mr. J. Senthil Kumar for his help with the maps.

The investigators of rotavirus vaccine impact assessment surveillance network are Sowmiya VS, Rama Prasad GS, Goru Krishna Babu, Padmalatha Pamu, B Manohar, Subal Pradhan, Mrutunjay Dash, Nirmal Kumar Mohakud, Rajib Ray, Geetha Gathwala, Suraj Chawla, Manoj Rawal, Madhu Gupta, Sanjeev S Choudhary, Shyam Kaushik, S Balasubramaniyan, CP Girish Kumar, Sridevi A Narayan, Kulaindaivel S, Anna Simon, R K Gupta, Suresh Goyal, Pramod Sharma, Sharad Thora, Pawan Ghanghoriya, Koshy George, Jayanta Goswami, Ashish Wakhlu, Vineeta Gupta, Mahima Mithal, Vipin Vashishtha.

REFERENCES

1 Parashar UD, Gibson CJ, Bresee JS, *et al.* Rotavirus and severe childhood diarrhea. *Emerg Infect Dis* 2006;**12**:304–6. doi:10.3201/eid1202.050006

2 World Health Organization. *World health statistics 2018: monitoring health for the SDGs*. 2018. http://apps.who.int/iris/bitstream/handle/10665/272596/9789241565585-eng.pdf?ua=1 (accessed 29 Nov 2018).

3 Tate JE, Burton AH, Boschi-Pinto C, *et al.* Global, Regional, and National Estimates of Rotavirus Mortality in Children <5 Years of Age, 2000-2013. *Clin Infect Dis Off Publ Infect Dis Soc Am* 2016;**62 Suppl 2**:S96–105. doi:10.1093/cid/civ1013

4 Troeger C, Forouzanfar M, Rao PC, *et al.* Estimates of global, regional, and national morbidity, mortality, and aetiologies of diarrhoeal diseases: a systematic analysis for the Global Burden of Disease Study 2015. *Lancet Infect Dis* 2017;**17**:909–48. doi:10.1016/S1473-3099(17)30276-1

5 Kotloff KL, Nataro JP, Blackwelder WC, *et al.* Burden and aetiology of diarrhoeal disease in infants and young children in developing countries (the Global Enteric Multicenter Study, GEMS): a prospective, case-control study. *Lancet Lond Engl* 2013;**382**:209–22. doi:10.1016/S0140-6736(13)60844-2

For peer review only - http://bmjopen.bmj.com/site/about/guidelines.xhtml

6 Liu J, Platts-Mills JA, Juma J, *et al.* Use of quantitative molecular diagnostic methods to identify causes of diarrhoea in children: a reanalysis of the GEMS case-control study. *Lancet Lond Engl* 2016;**388**:1291–301. doi:10.1016/S0140-6736(16)31529-X

7 John J, Sarkar R, Muliyil J, *et al.* Rotavirus gastroenteritis in India, 2011–2013: Revised estimates of disease burden and potential impact of vaccines. *Vaccine* 2014;**32**:A5–9. doi:10.1016/j.vaccine.2014.03.004

8 Esposito DH, Tate JE, Kang G, *et al.* Projected Impact and Cost-Effectiveness of a Rotavirus Vaccination Program in India, 2008. *Clin Infect Dis* 2011;**52**:171–7. doi:10.1093/cid/ciq094

9 Neogi SB, Hasan H, Sheikh K, *et al.* Scope for rotavirus vaccination in India: revisiting the scientific evidence. *Indian J Pediatr* 2011;**78**:1251–5. doi:10.1007/s12098-011-0448-3

10 Kang G, Arora R, Chitambar SD, *et al.* Multicenter, hospital-based surveillance of rotavirus disease and strains among indian children aged <5 years. *J Infect Dis* 2009;**200 Suppl 1**:S147-153. doi:10.1086/605031

11 National Rotavirus Surveillance Network, Kumar CPG, Venkatasubramanian S, *et al.* Profile and Trends of Rotavirus Gastroenteritis in Under 5 children in India, 2012 - 2014, Preliminary Report of the Indian National Rotavirus Surveillance Network. *Indian Pediatr* 2016;**53**:619–22.

12 Mullick S, Mandal P, Nayak MK, *et al.* Hospital based surveillance and genetic characterization of rotavirus strains in children (<5 years) with acute gastroenteritis in Kolkata, India, revealed resurgence of G9 and G2 genotypes during 2011-2013. *Vaccine* 2014;**32 Suppl 1**:A20-28. doi:10.1016/j.vaccine.2014.03.018

13 S. Namjoshi G, Mitra M, K. Lalwani S, *et al.* Rotavirus gastroenteritis among children less than 5 years of age in private outpatient setting in urban India. *Vaccine* 2014;**32**:A36–A44. doi:10.1016/j.vaccine.2014.03.070

14 Ruiz-Palacios GM, Pérez-Schael I, Velázquez FR, *et al.* Safety and efficacy of an attenuated vaccine against severe rotavirus gastroenteritis. *N Engl J Med* 2006;**354**:11–22. doi:10.1056/NEJMoa052434

15 Vesikari T, Matson DO, Dennehy P, *et al.* Safety and efficacy of a pentavalent human-bovine (WC3) reassortant rotavirus vaccine. *N Engl J Med* 2006;**354**:23–33. doi:10.1056/NEJMoa052664

16 Rotavirus vaccines. WHO position paper – January 2013. *Releve Epidemiol Hebd* 2013;**88**:49–64.

17 Panda S, Das A, Samanta S. Synthesizing evidences for policy translation: a public health discourse on rotavirus vaccine in India. *Vaccine* 2014;**32 Suppl 1**:A162-170. doi:10.1016/j.vaccine.2014.03.037

For peer review only

For peer review only - http://bmjopen.bmj.com/site/about/guidelines.xhtml

18 Rose J, Hawthorn RL, Watts B, *et al.* Public health impact and cost effectiveness of mass vaccination with live attenuated human rotavirus vaccine (RIX4414) in India: model based analysis. *BMJ* 2009;**339**:b3653.

19 Bhandari N, Rongsen-Chandola T, Bavdekar A, *et al.* Efficacy of a monovalent human-bovine (116E) rotavirus vaccine in Indian infants: a randomised, double-blind, placebo-controlled trial. *Lancet Lond Engl* 2014;**383**:2136–43. doi:10.1016/S0140-6736(13)62630-6

20 Mehendale S, Venkatasubramanian S, Girish Kumar CP, *et al.* Expanded Indian National Rotavirus Surveillance Network in the Context of Rotavirus Vaccine Introduction. *Indian Pediatr* 2016;**53**:575–81.

21 Shri J P Nadda launches Rotavirus vaccine as part of Universal Immunization Programme; terms it a "historic moment." http://pib.nic.in/newsite/PrintRelease.aspx?relid=138342 (accessed 29 Nov 2018).

22 Shri JP Nadda launchesExpansion of Rotavirus vaccine under Universal Immunization Programme. http://pib.nic.in/newsite/PrintRelease.aspx?relid=158549 (accessed 29 Nov 2018).

23 India: WHO & UNICEF estimates of immunization coverage: 2017 revision. http://www.who.int/immunization/monitoring_surveillance/data/ind.pdf (accessed 25 Oct 2018).

24 Ichihara MYT, Rodrigues LC, Teles Santos CAS, *et al.* Effectiveness of rotavirus vaccine against hospitalized rotavirus diarrhea: A case-control study. *Vaccine* 2014;**32**:2740–7. doi:10.1016/j.vaccine.2014.01.007

25 Velázquez RF, Linhares AC, Muñoz S, *et al.* Efficacy, safety and effectiveness of licensed rotavirus vaccines: a systematic review and meta-analysis for Latin America and the Caribbean. *BMC Pediatr* 2017;**17**. doi:10.1186/s12887-016-0771-y

26 Patel MM, Parashar UD. Assessing the effectiveness and public health impact of rotavirus vaccines after introduction in immunization programs. *J Infect Dis* 2009;**200 Suppl 1**:S291-299. doi:10.1086/605059

27 Lamberti LM, Ashraf S, Walker CLF, *et al.* A Systematic Review of the Effect of Rotavirus Vaccination on Diarrhea Outcomes Among Children Younger Than 5 Years. *Pediatr Infect Dis J* 2016;**35**:992–8. doi:10.1097/INF.0000000000001232

28 Reddy S, Nair NP, Giri S, *et al.* Safety monitoring of ROTAVAC vaccine and etiological investigation of intussusception in India: study protocol. *BMC Public Health* 2018;**18**:898. doi:10.1186/s12889-018-5809-7

29 Kliegman R, Behrman RE, Nelson WE, editors. *Nelson textbook of pediatrics*. Edition 20. Phialdelphia, PA: : Elsevier 2016.

For peer review only

For peer review only - http://bmjopen.bmj.com/site/about/guidelines.xhtml

30 Ruuska T, Vesikari T. Rotavirus disease in Finnish children: use of numerical scores for clinical severity of diarrhoeal episodes. *Scand J Infect Dis* 1990;**22**:259–67. doi:10.3109/00365549009027046

31 Tate JE, Patel MM, Cortese MM, *et al.* Use of Patients With Diarrhea Who Test Negative for Rotavirus as Controls to Estimate Rotavirus Vaccine Effectiveness Through Case-Control Studies. *Clin Infect Dis Off Publ Infect Dis Soc Am* 2016;**62 Suppl 2**:S106-114. doi:10.1093/cid/civ1014

32 Meridian Bioscience, Inc. Premier Rotaclone. http://www.meridianbioscience.com/diagnostic-products/rotovirus-and-adenovirus/premier/premier-rotaclone.aspx (accessed 18 Feb 2019).

33 Haber M, Lopman BA, Tate JE, *et al.* A comparison of the test-negative and traditional case-control study designs with respect to the bias of estimates of rotavirus vaccine effectiveness. *Vaccine* 2018;**36**:5071–6. doi:10.1016/j.vaccine.2018.06.072

34 Government of India. Ministry of Health and Family Welfare. Mission Indradhanush. http://pib.nic.in/newsite/PrintRelease.aspx?relid=181816 (accessed 25 Oct 2018).

35 Bines JE, Ivanoff B, Justice F, *et al.* Clinical case definition for the diagnosis of acute intussusception. *J Pediatr Gastroenterol Nutr* 2004;**39**:511–8.

36 Kang G, Desai R, Arora R, *et al.* Diversity of circulating rotavirus strains in children hospitalized with diarrhea in India, 2005-2009. *Vaccine* 2013;**31**:2879–83. doi:10.1016/j.vaccine.2013.04.030

37 Babji S, Arumugam R, Sarvanabhavan A, *et al.* Multi-center surveillance of rotavirus diarrhea in hospitalized children <5 years of age in India, 2009-2012. *Vaccine* 2014;**32 Suppl 1**:A10-12. doi:10.1016/j.vaccine.2014.03.001

38 Palma O de, Cruz L, Ramos H, *et al.* Effectiveness of rotavirus vaccination against childhood diarrhoea in El Salvador: case-control study. undefined. 2010./paper/Effectiveness-of-rotavirus-vaccination-against-in-Palma-Cruz/a4eac970bf37d83d9d841d4b8351415ed2f86bad (accessed 25 Oct 2018).

39 Justino MCA, Linhares AC, Lanzieri TM, *et al.* Effectiveness of the monovalent G1P[8] human rotavirus vaccine against hospitalization for severe G2P[4] rotavirus gastroenteritis in Belém, Brazil. *Pediatr Infect Dis J* 2011;**30**:396–401. doi:10.1097/INF.0b013e3182055cc2

40 The Anganwadi Workers of India – Connecting for Health, Education, Childcare at the Grassroots Level. http://healthopine.com/the-anganwadi-workers-of-india-connecting-for-health-at-the-grassroots/ (accessed 20 Mar 2018).

41 Parashar UD, Johnson H, Steele AD, *et al.* Health Impact of Rotavirus Vaccination in Developing Countries: Progress and Way Forward. *Clin Infect Dis Off Publ Infect Dis Soc Am* 2016;**62 Suppl 2**:S91-95. doi:10.1093/cid/civ1015

For peer review only - http://bmjopen.bmj.com/site/about/guidelines.xhtml

Figure 1: Location of surveillance sites in states introducing Rotavac into the universal immunization programme

Legends: 1 Kurnool, 2 Kakinada, 3 Vishakhapatnam, 4 Tirupati, 5 Cuttack 6, 7, 8 - Bhubaneswar, 9 Rohtak, 10 Mewat, 11 Sonipat, 12 Shimla, 13 Tanda, 14 Chandigarh, 15, 16- Chennai, 17 Madurai, 18, 19, 20, 21- Vellore, 22 Jaipur, 23 Udaipur, 24 Jodhpur, 25 Indore, 26 Jabalpur, 27 Tezpur, 28 Guwahati, 29 Lucknow, 30 Varanasi, 31 Gorakhpur, 32 Bijnor

Figure 2: Process for establishment of a new surveillance site

For peer review only

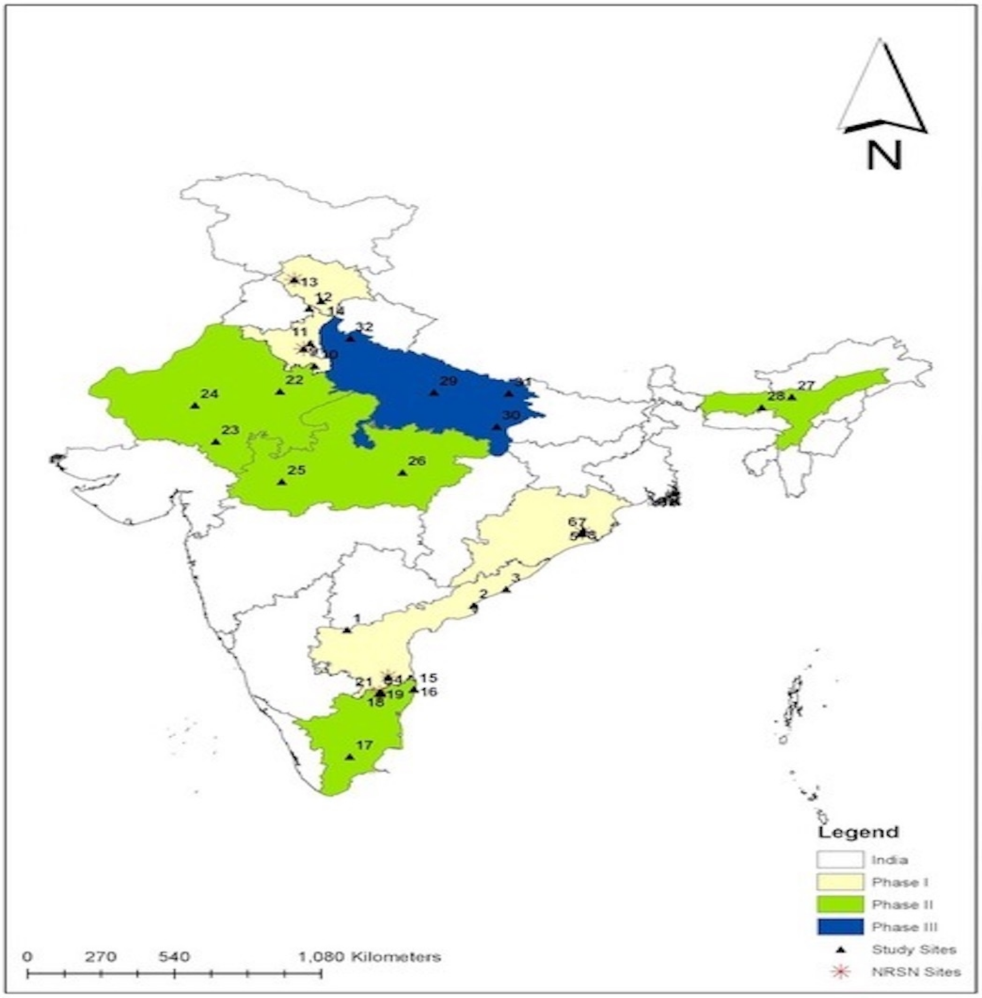

Figure 1: Location of surveillance sites in states introducing Rotavac into the Universal Immunization Programme

90x90mm (300 x 300 DPI)

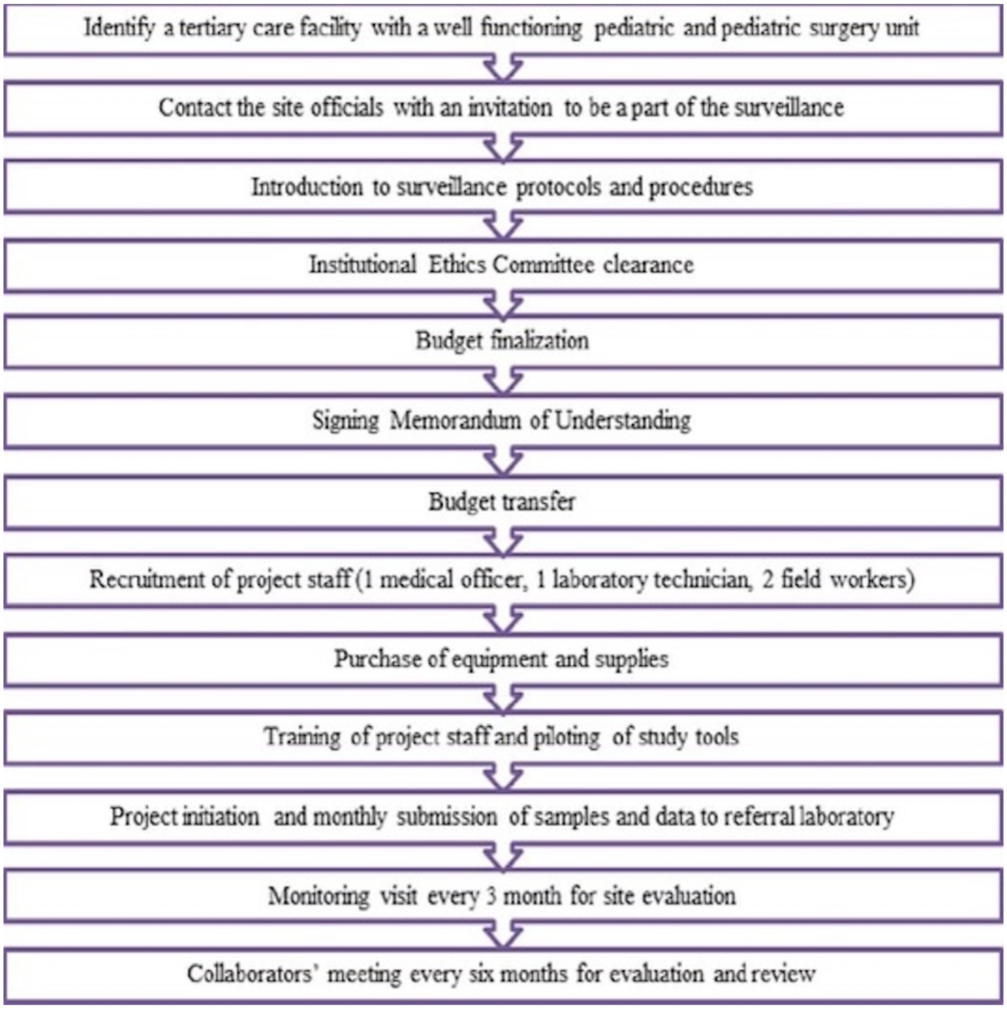

Figure 2: Process of establishment of a new surveillance site

90x90mm (300 x 300 DPI)

SupplementaryTable1. Baseline data from sentinel surveillance sites included in the impact assessment project

Sl. no.	Name of surveillance site	No. of under- five AGE admissions a year#
1	Kurnool Medical College, Kurnool, Andhra Pradesh	410
2	Government General hospital, Kakinada, Andhra Pradesh	400
3	King George Hospital, Vishakhapatnam, Andhra Pradesh	300
4	Sri Venkateswara Medical College, Tirupati, AP	400
5	Sardar Vallabhai Patel Post Graduate Institute of Pediatrics, Cuttack, Odisha	720
6	Kalinga Institute of Medical Sciences, Bhubaneshwar, Odisha	340
7	Institute of Medical Sciences and SUM Hospital, Bhubaneshwar, Odisha	335
8	Hi-Tech Hospital, Bhubaneshwar, Odisha	490
9	Pandit Bhagwat Dayal Sharma Post Graduate Institute of Medical Sciences, Rohtak, Haryana	250
10	Shaheed Hasan Khan Mewati Government Medical College, Mewat, Haryana	620
11	BPS Government Medical College for Women, Sonipat, Haryana	320
12	Indira Gandhi Government Medical College, Shimla, HP	200
13	Rajendra Prasad Government Medical College, Tanda, HP	300
14	Post Graduate Institute of Medical Education and Research, Chandigarh	600

For peer review only

15	The Institute of Child Health and Hospital for Children, Chennai, Tamil Nadu	488
16	Kanchi Kama Koti Child Trust Hospital, Chennai, Tamil Nadu	528
17	Government Medical College, Madurai, Tamil Nadu	110
18	Christian Medical College, Vellore, Tamil Nadu	
19	Nalam Hospital, Vellore, Tamil Nadu	
20	Narayani Hospital and Research Centre, Vellore, Tamil Nadu	
21	GVMC, Adukkumparai, Tamil Nadu	
22	Sawai Man Singh Medical College, Jaipur, Rajasthan	1763
23	Dr. Sampurnanand Medical College, Jodhpur, Rajasthan	1627
24	Rabindranath Tagore Medical college, Udaipur, Rajasthan	1660
25	King George Medical College, Lucknow, Uttar Pradesh	244
26	Institute of Medical Sciences, Banaras Hindu University, Varanasi, Uttar Pradesh	144
27	BRD Medical College, Gorakhpur, Uttar Pradesh	600
28	Mangala Hospital and Research Centre, Bijnor, Uttar Pradesh	550
29	NSCB Medical College, Jabalpur, Madhya Pradesh	700
30	Mahatma Gandhi Memorial Medical college, Indore, Madhya Pradesh	710
31	Government Medical college, Guwahati, Assam	560
32	Baptist Christian Hospital, Tezpur, Assam	232

#Retrospective data for the year 2015

For peer review only

Supplementary Table 2: Ethical committee details for Protocol submission

Sl. No.	Name of the institution	Name of the ethical committee (Reference number)
1	Kurnool Medical College, Kurnool, Andhra Pradesh	Institutional Ethical committee of Kurnool Medical College, Kurnool dated 12.11.2015
2	Government General Hospital, Kakinada, Andhra Pradesh	Institutional Ethics Committee cell of Rangaraya Medical College (IEC/RMC/2017/241 dated 06.05.2017)
3	Andhra Medical College, Vishakhapatnam, Andhra Pradesh	Institutional Ethical committee of King George Hospital, Vishakhapatnam (proposal 1 dated 19.11.2016)
4	Sri Venkateswara Medical College, Tirupati, Andhra Pradesh	Institutional Ethical committee of S.V Medical College (Lr. No. 2/2016 dated 07.02.2016)
5	Sardar Vallabhai Patel Post Graduate Institute of Pediatrics, Cuttack, Odisha	Institutional Ethical committee of SCB Medical College, Cuttack (IEC no. 273 dated 4.4.2016)
6	Kalinga Institute of Medical Sciences, Bhubaneswar, Odisha	Ethics committee of Kalinga Institute of Medical Sciences, Bhubaneswar (KIMS/Dir/11/16 dated 6.01.2016)
7	Institute of Medical Sciences and SUM Hospital, Bhubaneswar, Odisha	Institutional Ethical Committee of Institute of Medical Sciences and SUM Hospital, Bhubaneswar (Letter no. 232/20/11/2015 dated 20.11.2015)
8	Hi-Tech Hospital and Medical college, Bhubaneswar, Odisha	Institutional Ethical Committee of Hi-Tech Medical College and Hospital (HMCH/IEC/15/3067 dated 29.12.2015)
9	Pandit Bhagwat Dayal Sharma Post Graduate Institute of Medical Sciences, Rohtak, Haryana	Institutional Ethics Committee, Pandit Bhagwat Dayal Sharma Post Graduate Institute of

For peer review only

		Medical Sciences, Rohtak (No.IEC/16/41 dated 28.03.2016)
10	Shaheed Hasan Khan Mewati Government Medical College, Mewat, Haryana	Institutional Ethics Committee, Shaheed Hasan Khan Mewati Government Medical College, Mewat (SHKM/CM/2016/890 dated 13.02.2016)
11	BPS Government Medical College for Women, Sonipat, Haryana	Institutional Ethics Committee, BPS Government Medical College for Women, Sonipat (No. Peds/BPSGMC/16 dated 01.04.2016)
12	Post Graduate Institute of Medical Education and Research, Chandigarh	Institutional Ethics Committee, Post Graduate Institute of Medical Education and Research, Chandigarh (PGI/IEC/2016/3267 dated 03.08.2016)
13	Rajendra Prasad Government Medical College, Tanda, Himachal Pradesh	Institutional Ethics Committee, Rajendra Prasad Government Medical College, Tanda (HFW-H-DRPGMC/Ethics/2016 dated 21.01.2016)
14	Indira Gandhi Government Medical College, Shimla, Himachal Pradesh	Institutional Ethics Committee, Indira Gandhi Government Medical College, Shimla (ECR/533/Inst/HP/2014 dated 13.05.2016)
15	Kanchi Kama Koti Child Trust hospital, Chennai, Tamil Nadu	Ethics committee of Kanchi Kama Koti Childs Trust Hospital and the Childs Trust (CTMRF/EC/S/02/2017 dated 18.03.2017)
16	Institute of Child health, Egmore, Tamil Nadu	Institutional Ethics Committee of Madras Medical College, Chennai (No. 01052017 dated 02.05.2017)

For peer review only

17	Government Medical College, Madurai, Tamil Nadu	Ethics Committee of Madurai Medical College, Madurai (dated 27.11.2017)
18	Christian Medical College, Vellore, Tamil Nadu	Institutional Review Board, Christian Medical College, Vellore (IRB Min. no. 9530 dated 22.07.2015)
19	Government Vellore Medical College, Vellore, Tamil Nadu	Institutional Review Board, Christian Medical College, Vellore (IRB Min. no. 9530 dated 22.07.2015)
20	Nalam Hospital, Vellore, Tamil Nadu	Institutional Review Board, Christian Medical College, Vellore (IRB Min. no. 9530 dated 22.07.2015)
21	Narayani Hospital and Research Centre, Vellore, Tamil Nadu	Institutional Review Board, Christian Medical College, Vellore (IRB Min. no. 9530 dated 22.07.2015)
22	Sawai Man Singh Medical college, Jaipur, Rajasthan	Office of the Ethics Committee, Sawai Man Singh Medical college, Jaipur (3205/MC/EC/2017 dated 08.06.2017)
23	Rabindranath Tagore Medical College, Udaipur, Rajasthan	Ethics Committee, Rabindranath Tagore Medical College, Udaipur (RNT/STAT/IEC/2017/54 dated 18.04.2017)
24	Dr. Sampurnanand Medical College, Jodhpur, Rajasthan	Institutional Ethics Committee, Dr. Sampurnanand Medical College, Jodhpur (No.F.1/Acad/MC/JU/17/16277 dated 20.06.2017)
25	Mahatma Gandhi Memorial Medical College, Indore, Madhya Pradesh	Institutional Ethics Committee, Mahatma Gandhi Memorial

For peer review only

		Medical College, Indore (dated 24.05.2017)
26	Netaji Subhash Chandra Bose Medical College, Jabalpur, Madhya Pradesh	Institutional Review Board, Netaji Subhash Chandra Bose Medical College, Jabalpur, Madhya Pradesh
27	Baptist Christian Hospital, Tezpur, Assam	Institutional Ethics Committee, Emmanuel Hospital Association, New Delhi (Protocol No. 163 dated 23.03.2018)
28	Government Medical college, Guwahati, Assam	Institutional Review Board, Government Medical college, Guwahati (dated 28.02.2018)
29	King George Medical College, Lucknow, Uttar Pradesh	Institutional Ethics Committee, King George Medical University, Lucknow (No.515/Ethics/R-cell-17 dated 26.05.2017)
30	Institute of Medical Sciences, Banaras Hindu University, Varanasi, Uttar Pradesh	Institutional Ethics Committee, Institute of Medical Sciences, Banaras Hindu University, Varanasi (Dean/2016-17/EC/734 dated 31.03.2017)
31	Baba Ram Das Medical College, Gorakhpur, Uttar Pradesh	Institutional Review Board, Baba Ram Das Medical College, Gorakhpur, Uttar Pradesh (dated 20.07.2018)
32	Mangala Hospital and Research Centre, Bijnor, Uttar Pradesh	Institutional Ethics Committee, Mangala Hospital and Research Centre, Bijnor (IEC/MH/2018/01 dated 24.01.2018)

For peer review only

Supplementary table 3: Monitoring checklist for sites doing under five diarrhea surveillance

SITE PROFILE

1. Staff

Name of staff
Position Title or Duties
Full-time or Part-time for rotavirus responsibilities
Percentage of time spent working on rotavirus
Years of experience in Site
Undergone training at CMC or at site

2. Staff and equipment

Designated place for project staff
Storage of records (ICF/CRF etc.)
Refrigerator for storage of stool samples

3. Project management and supervision

Lines of supervision and accountability clear to all staff
Written protocols are available, and arrangements are in place for periodic review and evaluation
Arrangements are made for back-up staff to perform work during staff absences

PROJECT ACTIVITIES

1. Recording of recruitment

Number of children admitted for gastroenteritis
Source from which the details in the hospital logbook like ward registers, online information, emergency records etc.
How many locations are children recruited from?

For peer review only

Number of children enrolled for the project so far (Informed consent form completed)
Reasons for non-enrolment
Number of children for whom case report form was filled
Reasons for non-completion of CRF
Number of children for whom stool sample was collected
Reasons for non-collection of stool sample
Problems encountered by the site in completing this information

2. Review of informed consent forms

Number of ICFs reviewed
Location of filing the forms
Legibility of the consent forms especially name, date and signature
Signatures of parents/guardians
Signature of the investigator
Problems encountered by the site in completing this information

3. Completion of Case Report Forms

Number of CRFs reviewed
Number of CRFs with missing data fields
Signature and a date for completion of CRF
Record of stool sample collection
Verification of stool sample collection against the hospital logbook
Verification of stool sample collection against the sample transmittal log

For peer review only

Problems encountered by the site in completing this information

4. Collection of vaccination information

What are the sources of vaccination information in the CRFs
Verification of dates on the CRFs from the source
Problems encountered by the site in completing this information

5. Collection of stool samples

Collection of stool samples
Number of samples collected
Labelling of the containers
Storage of samples in the deep freezer including temperature maintenance and positioning of the samples
Quantity of the collected samples
Verification of stool samples against the hospital logbook
Verification of stool samples against the CRF
Verification of stool sample collection against the sample transmittal log
Problems encountered by the site in completing this information

SUMMARY INDICATORS

Enrolment of >90% of admitted cases of gastroenteritis
Collection of adequate stools from >90% of enrolled children
Complete documentation (ICF/CRF) in >90% of enrolled children
Collection of vaccination record (patient retained/government) for >75% of enrolled children

For peer review only
